# Draft genome sequence of first monocot-halophytic species *Oryza coarctata* reveals stress-specific genes

**DOI:** 10.1038/s41598-018-31518-y

**Published:** 2018-09-12

**Authors:** Tapan Kumar Mondal, Hukam Chand Rawal, Soni Chowrasia, Deepti Varshney, Alok Kumar Panda, Abhishek Mazumdar, Harmeet Kaur, Kishor Gaikwad, Tilak Raj Sharma, Nagendra Kumar Singh

**Affiliations:** ICAR-National Research Centre on Plant Biotechnology, Pusa, New Delhi 110012 India

## Abstract

*Oryza coarctata* (KKLL; 2n = 4x = 48, 665 Mb) also known as *Porteresia coarctata* is an extreme halophyte species of genus *Oryza*. Using Illumina and Nanopore reads, we achieved the assembled genome size of 569.9 Mb, accounting 85.69% of the estimated genome size with N50 of 1.85 Mb and 19.89% repetitive region. We also found 230,968 simple sequence repeats (SSRs) and 5,512 non-coding RNAs (ncRNAs). The functional annotation of predicted 33,627 protein-coding genes and 4,916 transcription factors revealed that high salinity adaptation of this species is due to the exclusive or excessive presence of stress-specific genes as compared to rice. We have identified 8 homologs to salt-tolerant *SOS1* genes, one of the three main components of salt overly sensitive (*SOS*) signal pathway. On the other hand, the phylogenetic analysis of the assembled chloroplast (134.75 kb) and mitochondrial genome (491.06 kb) favours the conservative nature of these organelle genomes within *Oryza* taxon.

## Introduction

Soil salinity is a major abiotic stress reducing the yield of rice cultivated globally and is increasing gradually due to surface irrigation, high cropping intensity and cultivation of high yielding variety^1^. In general, rice is a glycophyte species except few tolerant landraces which can grow up to a maximum limit of 12 E.Ce (electrical conductivity) dS/m^2^. Those landraces have been extensively used by the plant breeders to identify salt tolerant QTLs for introgression to popular salt sensitive genotypes of rice^3^. Thus genetic potential for salt tolerance among the existing population has been fully exploited. Therefore, alternative effectual alleles from wild species may aid in amending the salinity tolerance to an advanced level. Wild species are a potential alternative source of many useful alleles that may not be present in the primary gene pool of the domesticated species.

*O*. *coarctata*, an allo-tetraploid species (chromosome number of 2n = 4X = 48, KKLL genome)^4,5^ is popularly known as Asian wild rice, grows naturally in the coastal region of South-East Asian countries, where this plant experiences the lunar tide and is exposed to submerge saline sea water in every alternative 12 h. It set flowers and seeds under high saline conditions of upto 40 E.CedS m^−1^ saline soil^6^. It is the only halophytic species in the genus *Oryza* and represents the evolutionary transition of aquatic plant to land plant because of the presence of ancient as well as advanced characteristics of terrestrial habitat^7^. Although several pinitol biosynthesis pathway genes individually have been cloned for functional genomics study^8^ but there is lack of large scale genomics resources for this species except one study each on transcriptome^9^ and on microRNA (miRNAs)^10^. Apart from these, some BAC end sequences and repeat libraries for *O*. *coarctata* were also reported as part of The Oryza Map Alignment Project (OMAP)^11–13^ but no efforts seems to be reported to decode the genome assembly of *O*. *coarctata*, which is the pre-requisite to harness its genetic potential for salinity tolerance. There are 24 morphologically distinct species in the genus *Oryza*, yet 11 of them have been sequenced till today though none of them are halophyte in nature. Genome sequence has several advantages such as i) generation of large number of markers which in turn help in marker assistant breeding, ii) revealed several genomic resources both for coding and non-coding region and hence can be used for evolutionary study by comparative genomics, iii) helps to identify the conserved and novel genes along with paralogs, hence providing better opportunity to select the gene of interest.

Most of the work done so far on the salinity genes have been employed on model but glycophytic species i.e. rice and *Arabidopsis* which have very less or no significance to endure high salinity. Recently, few halophytes have been sequenced such as *Thellungiella parvula*^14^, *T*. *salsuginea*^15^, *Eutrema salsugineum*^16^, but none of them are monocot in nature. Being a close wild relative of one of the most important cultivated crop world-wide i.e. rice, and monocot in nature, *O*. *coarctata* may aid to generate an important and large reservoir of salinity tolerant genes which, either through conventional breeding or through genetic engineering approaches, will help research community for improving salinity tolerance in rice plants.

In the present study, we have decoded the whole genome of a halophytic monocot species *O*. *coarctata* using Illumina and Nanopore reads to generate a high-quality (HQ) draft genome assembly of 569.9 Mb with a scaffold N50 of 1.85 Mb. We also assembled and reported the complete sequence of chloroplast and mitochondrial genome of *O*. *coarctata*. We discovered many stress-specific or salinity responsive genes, including *SOS1* (salt overly sensitive 1) genes, in *O*. *coarctata* which are either missing or present in low copy number in the rice. Evolutionary and syntenic studies have been performed at genome level, organelle level and gene level to unravel not only the conserved features of *O*. *coarctata* within *Oryza* genus but also to uncover its similarity with other halophytic species and differences from AA, BB and FF genome types of the genus *Oryza*. The genomic resources developed in this sequencing effort should contribute in advancing molecular breeding programmes against salinity and to investigate the *Oryza* genome evolution.

## Materials and Methods

### Genome size estimation

The plant, after collecting from the coastal region of Sundarban delta of West Bengal, India (21°36′N and 88° 15′E) was established at Net house through clonal propagation. To estimate the genome size, we used 20 mg leaf extract from a 10 cm long plant grown in pot. DNA content was estimated as fluorescence of propidium iodide (PI)-stained^17^ nuclei of *O*. *coarctata* genome using *Pisum sativum* (pea) (1 C = 9.09 pg) as an external standard^18^. Experiment was conducted with FACS cell sorter by BD-LSR II(BD-JH FACS Academy, Jamia Hamdard (Hamdard University) Hamdard Nagar, New Delhi, India)and data were analyzed by BD FACS Diva v.8.0.1 (http://www.bdbiosciences.com/in/instruments/software/facsdiva/features/overview.jsp). The whole experiment was repeated 3 times with 8 samples in each time.

### Library preparation and sequencing

Genomic DNA (gDNA) was isolated by CTAB method as per our previous protocol^2^ from young leaf of the same plant used to estimate the genome size. The quality of the isolated DNA was checked by a NanoDrop D-1000 spectrophotometer (NanoDrop Technologies, Wilmington, DE) and Qubit Fluorometer. This DNA was used to make one Illumina short Paired-end (PE) library of 151 bp long reads and four Mate-pair (MP)library such as 2 kb, 4 kb, 6 kb and 8 kb in size following the standard Illumina protocols (Illumina, San Diego, CA) and sequenced with HiSeq4000 platform (Illumina, San Diego, CA). In addition, we also used Nanopore long reads for better assembly which was sequenced on MinION Mk1b (Oxford Nanopore Technologies, Oxford, UK) using SpotON flow cell (R9.4) in a 48 h sequencing protocol on MinKNOW 1.4.32. Base calling was performed using Albacore and base called reads were processed using Poretools version 0.6.0^19^. All the sequencing works were carried out at M/S Genotypic Technology Private Limited, Bengaluru, India.

### *De-novo* assembly of nuclear genome

First, the raw reads were quality checked using FastQC_v.0.11.5 (https://www.bioinformatics.babraham.ac.uk/projects/fastqc/) and were further processed by PLATANUStrimmer^20^ for trimming the adaptors and low quality regions towards 3′-end at PHRED quality score cut-off of Q30. The processed paired-end (PE) reads were used for contig assembly using PLATANUS *de novo* assembler v1.2.4^20^ at default parameters. Scaffolding of the assembled contigs was performed using PLATANUS v1.2.4 and SSPACE (https://www.baseclear.com/genomics/bioinformatics/basetools/SSPACE, V3.0) with MP and Nanopore libraries followed by gap closing with GapCloser program (http://soap.genomics.org.cn/about.html) to fill the gaps within scaffolds.

Completeness of the assembled genome was evaluated by Core Eukaryotic Genes Mapping Approach (CEGMA) (http://korflab.ucdavis.edu/datasets/cegma/) and Benchmarking Universal Single-Copy Orthologs (BUSCO) (http://busco.ezlab.org/) with *O*. *sativa* ssp. *japonica* as the reference genome.

### Genome annotation of nuclear genome

Putative protein-coding genes were predicted using a combined strategy that integrates *ab intio* gene predictor AUGUSTUS 3.1 (http://bioinf.uni-greifswald.de/augustus/) and sequence evidence based annotation pipeline, MAKER-P v2.31.8 (http://www.yandell-lab.org/software/maker-p.html) with *O*. *sativa* ssp. *japonica* as reference gene model. In addition, 5 transcriptome SRA [SRX248542 (Salt450 along with Submerged); SRX248538 (Control); SRX248541 (Submerged); SRX248540 (Salt700); SRX248539 (Salt450)] were used as expression evidence for this prediction (Supplementary Table S1). Resulting gene models were filtered for valid start codon. The final set of predicted protein-coding genes was annotated with Blast2GO (version 4.01)^21^ by using BLAST^22^ based approach against a database containing functional plant genes downloaded from NCBI with an E-value cut-off of ≤1e^−10^. Genes with significant hits were assigned with GO (Gene Ontology) terms and EC (Enzyme Commission) numbers. InterProScan search and pathway analyses with KEGG database were also performed by using Blast2GO.

In order to predict the conserved and unique gene families among the *O*. *coarctata* genome and 11 other *Oryza* species, an HMMScan of HMMER 3.1 package (http://hmmer.org/) was run against the protein sequences of all these 12 species (Supplementary Table S1), based on the profiles compiled in Pfam release 31.0 (http://pfam.xfam.org/) of the Pfam database at default parameters.

### Prediction and analysis of F-box and SOS pathway genes

A combined approach of HMMER search and BLAST search was used to retrieve the putative F-box genes. F-box HMM domain (PF00646) was downloaded from Pfam site (http://pfam.xfam.org/). HMMER search and BLAST search was done against 33,627 protein sequences of *O*. *coarctata* with the E-value ≤ 1e^−10^. Both results were merged for non-redundant significant hits and were checked for the presence of F-box domain (PF00646) by CD search tool (https://www.ncbi.nlm.nih.gov/Structure/bwrpsb/bwrpsb.cgi).

A keyword based search was done for available *SOS1* genes at NCBI with keyword “plasma membrane Na^+^/H^+^ antiporter” or “*SOS1*” or “salt overly sensitive 1”. A non-redundant set of 149 *SOS1* genes was found in different plant species, ranging from a minimum length of 65 amino-acid to maximum 1213 amino-acid. Out of total 149 genes, 123 genes were found to have the “Na^+^/H^+^ antiporter or exchanger family” domain as predicted by CD-batch search tool. To predict the *SOS1* genes in *O*. *coarctata* genome, the 33627 genes of *O*. *coarctata* were BLAST searched against these 123 *SOS1* genes. The significant hits, with 60% identity, were checked for the presence of “Sodium/hydrogen exchanger family” domain (PF00999) at threshold of e-value ≤ 1e-^10^ by CD search tool. MEGA 7.0^23^ was used to perform alignment and phylogenetic analyses between the newly predicted *SOS1* genes in *O*. *coarctata* and known *SOS1* genes from other plant species.

### Identification of transcription factors

To identify the transcription factors (TFs) in *O*. *coarctata* genes, plant transcription factors sequences were downloaded from Plant Transcription Factor Database v4.0 (http://planttfdb.cbi.pku.edu.cn/) and a BLAST search based approach was used with cut-off values for E-value, identity and query-coverage as ≤1e^−10^, ≥40% and ≥50%, respectively^24^.

### Identification of repetitive elements and SSR markers

Repetitive elements, retrotransposons and DNA transposons were identified and masked in the assembled *O*. *coarctata* genome by using RepeatMasker Tool (http://www.repeatmasker.org/) against the RepBase v.20170127 (http://www.girinst.org/repbase/) using the reference genomic repeats of *O*. *sativa* for hard masking. LTR_FINDER v1.05 was used to identify full-length LTR (long terminal repeat) retrotransposon^25^. The SSRs were identified by MIcroSAtellite identification tool (MISA) perl script (http://pgrc.ipk-gatersleben.de/misa/). The minimum number of nucleotide repeats searched during the SSR analysis was set as ten for mono-, six for di-, four for tri- and three for tetra-, penta- and hexa-nucleotide repeats with maximal number of bases interrupting 2 SSRs in a compound microsatellite as 100.

### Identification of non-coding RNAs (ncRNA)

The ribosomal RNA (rRNA) and small nuclear RNA (snRNA) were identified by INFERNAL^26^ with default parameters against Rfam database (release 9.1) (http://rfam.xfam.org). To predict the miRNAs, we used two steps procedure: first, homology search against Rfam database with a cut-off of 90% identity and query coverage, secondly the presence of hairpin structure in the surrounding sequence of predicted miRNA. The tRNAscan-SE algorithms (Version 1.23)^27^ were used to identify and annotate transfer RNA (tRNA) genes with default parameters. For the prediction and annotation of small nucleolar RNA (snoRNA) genes in the assembled genome, snoScan was used with the yeast rRNA methylation sites and yeast rRNA sequences provided by the snoScan distribution^28^.

### Phylogenetic analysis with single-copy genes among *Oryza* species

All protein sequences of *O*. *coarctata* genome and 11 other sequenced *Oryza* species (Supplementary Table S1) were subjected to CD-HIT^29^ for clustering at cut-off of 90% coverage and similarity to form unique clusters. Clusters with a single copy gene from all 12 genomes, termed as Single-Copy-gene Clusters, were used for molecular phylogenetic analysis by using the steps as described by Kawano *et al*.^30^.

### Synteny analysis for conserved regions with other species

Conserved synteny is used as a measurement for evolutionary divergence^31^ or as calculation of conserved coding or non-coding region across different genomes^32^. To identify the conserved regions across genomes of interest, we performed the synteny analysis of the assembled genome of *O*. *coarctata* with the reference genome of *O*. *sativa* ssp. *japonica* and the model dicot species *A*. *thaliana*, along with its wild halophytic relatives *Schrenkiella parvula* and *E*. *salsugineum*. The genome sequences of these 4 species were downloaded from NCBI (Supplementary Table S1) and BLAST search was used to find out the conserved regions between *O*. *coarctata* assembled genome and these 4 reference genomes with an e-value cut-off of ≤1e^−10^. Homologous blocks of alignment length of ≥500 bp between the references and the assembled genome were rendered in the synteny plot generated using CIRCOS tool v0.67-7^33^.

### Reference-based assembly of chloroplast genome

We used the Nanopore long reads to assemble the chloroplast genome of *O*. *coarctata*. The Nanopore reads were BLAST searched against chloroplast genomes of 11 *Oryza* species of interest (Supplementary Table S1). Out of 1,717,607 Nanopore reads, 28,218 reads (95.26 Mb) were showing significant hits (E-value ≤ 1e^−10^) against *Oryza* species and were used for 2-round assembly by CLC Genomics Workbench 9.5.1 (CLC Bio, Arhus, Denmark) with chloroplast sequence of *O*. *sativa *ssp. *indica* and *japonica* as reference. Circular orientation was checked and chloroplast annotation was done by CpGAVAS^34^. The four junctions between 2 inverted repeat regions and 2 single copy regions were validated by PCR amplification using 4 pairs of primers (Supplementary Table S2). GenBank file was prepared with sequin and subjected to OGDraw v1.2^35^ to generate chloroplast gene map. We also performed the phylogenetic analysis of this assembled chloroplast genome along with chloroplast sequence of 11 other sequenced *Oryza* species. All 12 sequences were aligned by ClustalW and evolutionary analyses were conducted using the Maximum Likelihood method based on the Tamura-Nei model in MEGA7^23^.

### Reference based assembly of mitochondrial genome

The processed and quality filtered PE and MP reads were aligned against the mitochondrion genome of *O*. *sativa* ssp. *japonica* (NC_011033.1, 490,520 bp) using bowtie2-2.2.5 (http://bowtie-bio.sourceforge.net/index.shtml) and aligned reads were *de novo* assembled by Spades-3.9.1^36^. The scaffolding and gap closing were done with SSPACE and GapCloser, respectively. The assembled genome was annotated for genes and tRNAs by MITOS^37^ and MiTFi^38^, respectively. Phylogenetic analysis was done with 4 other available mitochondrial genomes of *Oryza* species (*O*. *sativa* ssp. *japonica, O*. *sativa* ssp. *indica, O*. *rufipogon* and *O*. *minuta*), 2 monocot species (*Triticum aestivum* and *Sorghum bicolor*) and 2 dicot species (*Glycine max* and *A*. *thaliana*) by MEGA7^23^.

### Accession Numbers

Project Information: NCBI BioProject ID: PRJNA396417. Chloroplast Genome Assembly: GenBank Accession no. MG383937. Mitochondrial genome Assembly: GenBank Accession no. MG429050.

## Results

### Genome sequencing, assembly and quality assessment

The genome size calculation for *O*. *coarctata* genome from young leaf tissue of pot-grown single plant using *P*. *sativum* as an external standard with flow cytometric method resulted in the estimated genome size of 665 Mb (Supplemental Fig. S1a,b)^39^. We have generated a total of 166.69 Gb of HQraw data comprising of short Illumina PEreads (123.78 Gb), MP libraries of 2 Kb (11.12 Gb), 4 Kb (3.06 Gb), 6 Kb (11.23 Gb) and 8 Kb (11.13 Gb) insert size, and Nanopore reads (6.35 Gb) to yield the genome-sequencing depth (x) of 250.66 (Supplemental Table S3). The average read length for Illumina reads were 151 bp, and that for long reads from Nanopore was 3,697 bp with GC% in the range of 42–44% for the sequencing reads generated from each of these technology. The nanopore technology, which was used to generate long reads, was quite successful as more than 60% of the reads having length above 1 Kb, including ~10% above 10 Kb length with maximum read length of 677.71 Kb (Supplemental Fig. S2a,b). The HQ Illumina PE reads were assembled into 101,992 contigs (555.66 Mb; N50 = 15.13 Kb) using PLATANUS followed by scaffolding with MP and Nanopore reads using SSPACE. The final assembly of 569.99 Mb covering 85.71% of the estimated genome size was obtained after filling the gaps within the scaffolds (Table 1). This final assembly contains 58,362 scaffolds with N50 and L50 values were 1,858,627 bp and 84, respectively. The long reads performed significantly well by reducing 101,992 contigs into 58,362 scaffolds and increased the N50 to ~122 times higher (15.13 Kb to 1.85 Mb). In this final assembly of 569.99 Mb, we achieved the longest contig size of 7.85 Mb with an average scaffold length of 9,766 bp.

**Table 1:** Summary of genome assembly, predicted genes and repeats statistics.

Assembly	
Genome-sequencing depth (X)	250.66
Estimated genome size (Mb)	665
Estimated coverage (%)	85.71
Number of Scaffolds	58,362
Total Length of Scaffolds (bp)	569,994,164
Longest Scaffold (bp)	7,855,609
Average Scaffold Length (bp)	9766.5
GC %	41.5
Total Number of Non-ATGC Characters	3,579,650
Percentage of Non-ATGC Characters	0.63
Scaffolds > = 1 Kbp	13840 (551.47 Mb)
Scaffolds > = 100 Kbp	411 (526.46 Mb)
Scaffolds > = 1 Mbp	181 (417.36 Mb)
Scaffolds > = 5 Mbp	12 (76.96 Mb)
N50 of Scaffolds (bp)	1,858,627
L50 of Scaffolds (bp)	84
Number of contigs	101,992
Total Length of Contigs (bp)	555,666,118
N50 of Contigs (bp)	15,132
Longest Contig (bp)	157,100
**Gene Annotation**	
Number of Gene Models	33,627
Total Span of Genes (bp)	38,572,471
Gene Length in the Genome (%)	6.77%
Average Gene Length (bp)	1,147
Largest Gene Length (bp)	14,926
Number of Genes annotated	26,569 (79.01%)
**Repeats**	
Masked Repeat Sequence Length (bp)	113,352,647
Repeat Sequences in the genome (%)	19.89

Both CEGMA and BUSCO were used to check the completeness of the assembled genome for the presence of core genes. Results from CEGMA, registered for 92.34% (97.18%, partial) completeness of the assembled genome with 229 of 248 ultra-conserved core eukaryotic genes (CEGs) were present in the genome (Supplemental Table S4). The genome completeness raised upto 98.70% when normalized with respect to reference genome of *O*. *sativa* spp. *japonica* Nipponbare-IRGSP-1.0. Similar results were found for the analysis with BUSCO that registered 97.08% completeness with normalized value of 99.43% (Supplemental Fig. S3).

### Gene prediction and annotation

With high level of completeness for the presence of core genes in the assembled genome, as predicted with CEGMA and BUSCO, we proceed with gene prediction and annotation in the assembled 58,362 scaffolds by integrating AUGUSTUS and MAKER-P annotation pipeline. A 3.32% (1,938) of the total scaffolds were found containing a total of 33,627 predicted protein-coding genes with length ranging from 180 bp to 1926 bp and an average length of 1,147 bp. These numbers are quite comparable with the predicted genes among the 11 other *Oryza* species sequenced so far (Supplemental Table S5).

**Figure 1:**
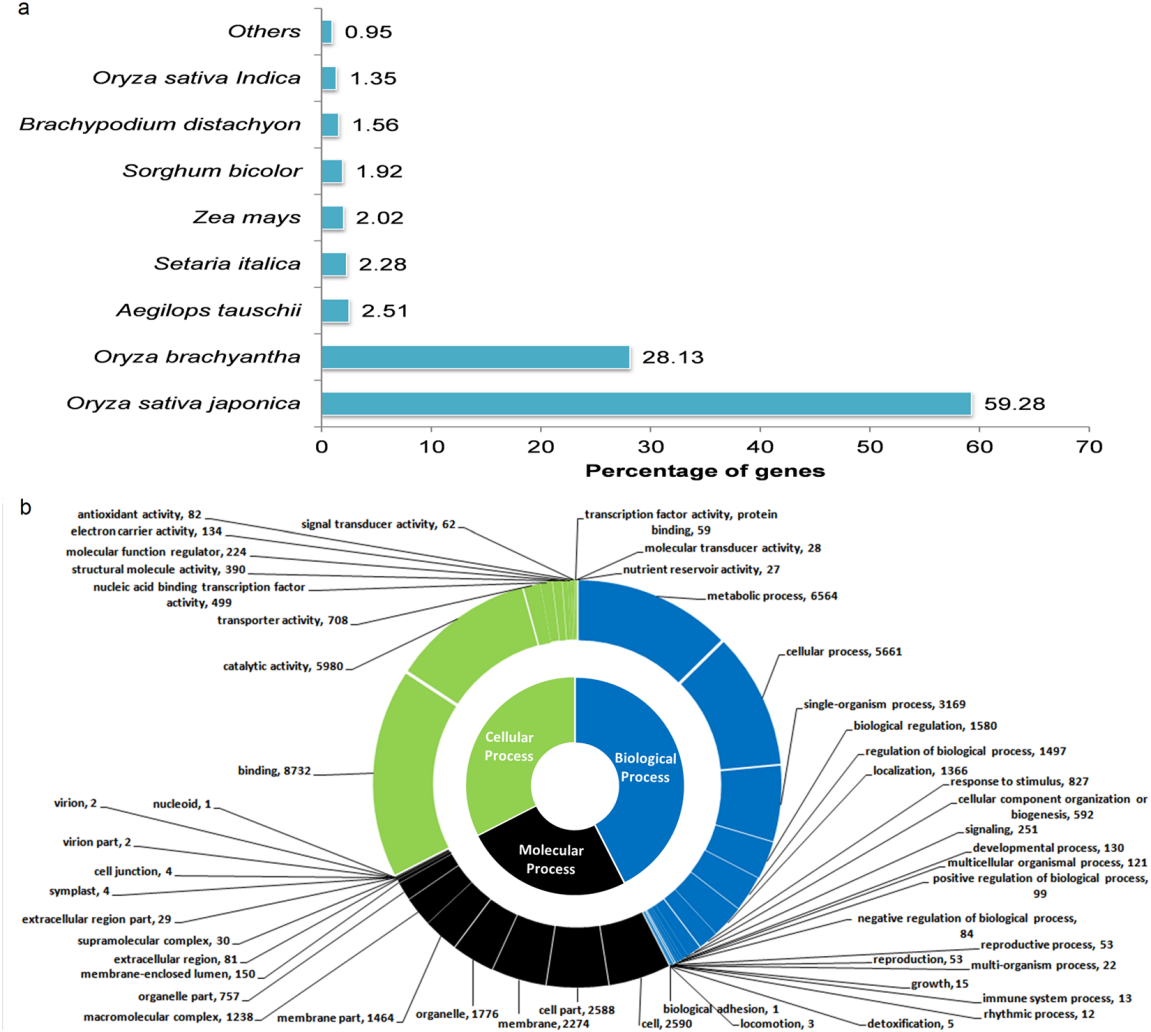
BLAST based annotation of predicted genes in *O*. *coarctata*. (**a**) Species distribution of 26,569 genes with BLAST hits showed that majority of them (i.e. 59.28%) had top BLAST hits against *O*. *sativa*ssp. *japonica*, followed by *O*. *brachyantha, Aegilop stauschii, Setaria italica* and *Zea mays* with28.13%, 2.15%, 2.27% and 2.01%, respectively, (**b**) GO terms distribution of 26,569 genes in level 2 GO categroization in biological Process, cellular component, and molecular function.

Of 33,627 predicted genes, a total number of 26,569 (79.01%) were assigned functions based on their significant best BLASTP hits to Plant Genes DB by using Blast2GO. Species distribution showed that majority (i.e. 59.28%) of these 26,569 genes had top BLAST hits against *O*. *sativa* ssp. *japonica*, followed by *O*. *brachyantha, Aegilops tauschii, Setaria italica* and *Zea mays* with 28.13%, 2.15%, 2.27% and 2.01%, respectively (Fig. 1a). In total, a good amount of 87.41% genes showed homology against rice and *O*. *brachyantha*, in spite of less number of genes from these genomes in the plant gene database used for annotation (Supplemental Table S6), suggesting its existence somewhere between AA and FF genome type or between domesticated and wild relatives. In order to identify the genes associated with biological processes, molecular functions and cellular process, we carried out the functional annotation in terms of Gene Ontology (GO) using BLASTP results. A total of 35,690 GO terms were assigned to 16,357 genes associated with biological processes, cellular components and molecular functions (Fig. 1b). Among the biological processes, the most prevalent were metabolic process (6,564), cellular process (5,661) and single-organism process (3,169) (Fig. 1b). Apart from these three generally high-scoring categories, there are 19 more groups with at least 1 or more genes with assigned GO terms but noticeable ones are “response to stimulus” (827 genes), “signalling” (251 genes) and “developmental process” (130 genes). The 827 genes under GO category “response to stimulus” were assigned with 207 GO terms or sub-categories like “response to stress”, “salt-stress”, “oxidative stress”, “water deprivation”, “auxin”, “abscisic acid”, “biotic-” and “abiotic-stimulus” pointing towards the significant presence of stress-related genes in the *O*. *coarctata* genome, which are either absent or present in very less number in rice (IRGSP 1.0) (Supplemental Table S7). For cellular processes associated genes, ~40% of genes were assigned GO terms for cell or cell-part category and ~29% genes to that for membrane or membrane-part category (Fig. 1b). With 51.59% (8,732 genes), the largest category among genes associated with GO terms for molecular functions was binding activity followed by catalytic activity (35.33%; 5,980 genes) (Fig. 1b). A total of 2,844 genes were annotated with enzyme code distribution in which high abundance of genes were assigned hydrolases enzyme (1336) and transferases (707) followed by oxidoreductase (404), lyases (155), isomerases (127) and ligases (115). Additionally, 4,640 of the predicted genes were functionally annotated with Kyoto Encyclopedia of Genes and Genomes (KEGG) database. The observed different types in abundance of protein function classes may be important to support different life-styles of the plant species. Finally, there were 7,058 genes in *O*. *coarctata* without any significant BLAST hits (E-value ≤ 1e^−10^) against plant genes database and 2,216 of these genes were found neither any significant GO hits nor any InterProScan match which accounts for 22.33% and 6.60% of total genes, respectively indicating that these could act as a gene-pool for *O*. *coarctata* specific and unique genes (Supplemental Table S8).

Transcription factors (TF), as key regulator for gene expression, are best suited for understanding the molecular mechanism and incorporating the abiotic stress tolerance in plants^40,41^. The BLAST based homology search of genes of *O*. *coarctata* against available plant TFs sequences in PlantTFDB 4.0 identified 4,916 genes (14.62% of total genes) in *O*. *coarctata* distributed in 56 different TF classes across 133 plant species (Supplemental Fig. S4, Table S9). These 4,916 TFs have a high abundance of the stress-response influencing TFs like MYB or MYB_related (519), NAC (322), bZIP (231), WRKY (177), HB (118), HSF (40), and AP (33). The identified 4,916 TF genes showed homology primarily against *O*. *punctata, O*. *sativa* ssp. *japonica, O*. *meridionalis, O*. *brachyantha, Malus domestica, O*. *sativa* ssp. *indica, Leersia perrieri, O*. *glumaepatula, O*. *nivara, O*. *longistaminata, Sisymbriumirio, Actinidia chinensis* and *Fragaria vesca* with over 100 genes against each of these plant species (Supplemental Table S9). About 40.30% (1,981) of the identified 8,135 TF genes were showing homology against 11 *Oryza* species.

**Figure 2:**
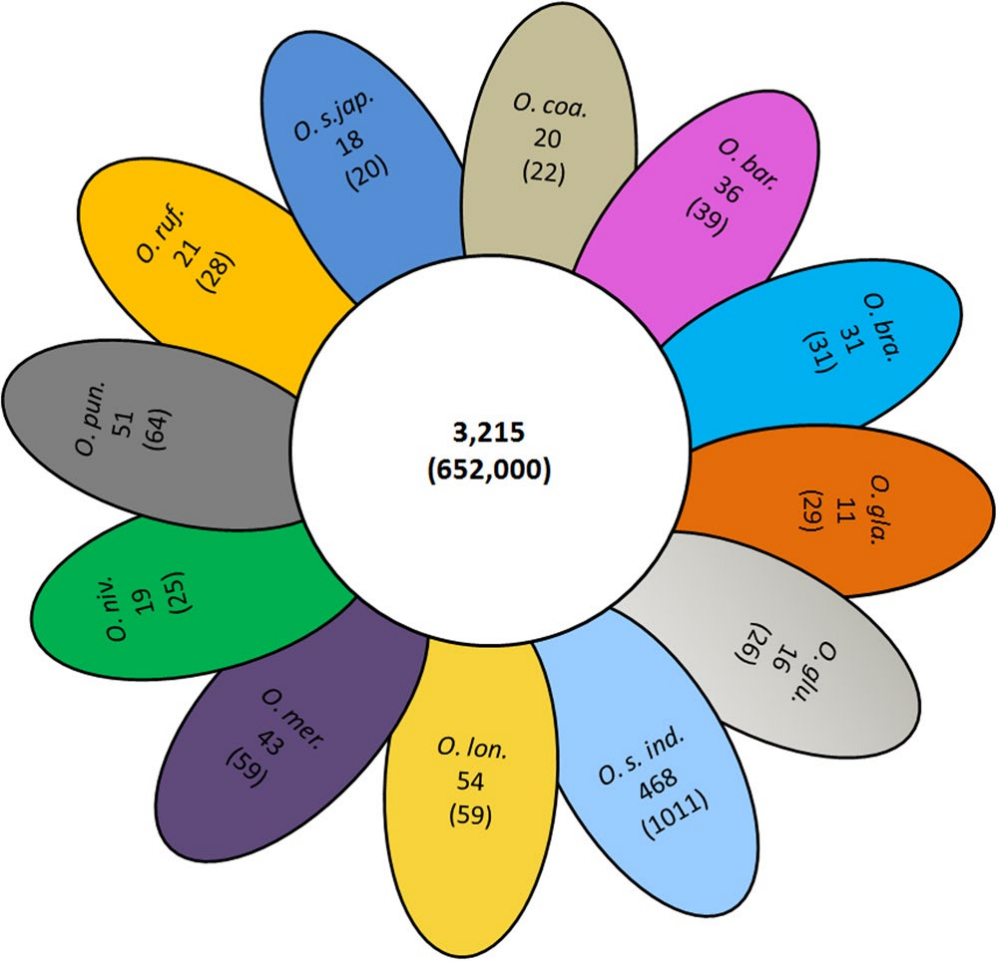
Number of shared and unique gene families among 12 *Oryza* species including *O*. *coarctata* genome with number of genes within families in parentheses showing a high number of shared genes (652,000) and families (3,215) among these *Oryza* species.

We compared the conserved and unique gene families between *O*. *coarctata* genome and 11 other sequenced *Oryza* species with representatives from *O*. *sativa* ssp. *japonica, O*. *sativa* ssp. *indica, O*. *rufipogon, O*. *punctata, O*. *nivara, O*. *meridionalis, O*. *longistaminata, O*. *glumaepatula, O*. *glaberrima, O*. *brachyantha* and *O*. *barthii*. This comparison resulted in a core set of 652,000 genes representing 3,215 clusters shared among all 12 *Oryza* species, encoding ancestral gene families (Fig. 2). We found a total of 20 gene clusters comprising 22 genes unique to the *O*. *coarctata* genome followed by *O*. *rufipogon* (21 unique gene families: 28 genes), *O*. *sativa* ssp. *japonica* (18 unique gene families: 20 genes), *O*. *glumaepatula* (16 unique gene families: 26 genes) and *O*. *glaberrima* (11 unique gene families: 29 genes). Interestingly, *O*. *sativa* ssp.*indica* had 468 unique gene families that contain 1,011 genes. The number of unique gene families clusters and gene in *O*. *longistaminata* (54 unique gene families: 59 genes)and *O*. *punctate* (51 unique gene families: 64 genes) genome were very similar followed by *O*. *meridionalis* (43 unique gene families: 59 genes).

F-box domain proteins are important for our analysis not only as one of the largest gene family controlling many biological functions but because of its important role in the regulation of various stress-response pathways and developmental processes in plants^42–45^. We identified 421 F-box genes in *O*. *coarctata* genome using HMMER and BLAST search based approach against F-box domain (PF00646). These F-box genes are about 1.25% of total genes in *O*. *coarctata* comparable to those with *A*. *thaliana* (2.53%), *V*. *vinifera* (0.51%), *P*. *trichocarpa* (0.81%), *O*. *sativa* (1.91%), *S*. *bicolor* (1.59%), *S*. *moellendorffii* (1.11%), and *P*. *patens* (0.42%), plant genomes representing different levels of plant kingdoms including a bryophyte, a lycophyte, monocots, and eudicots^46^.

**Figure 3:**
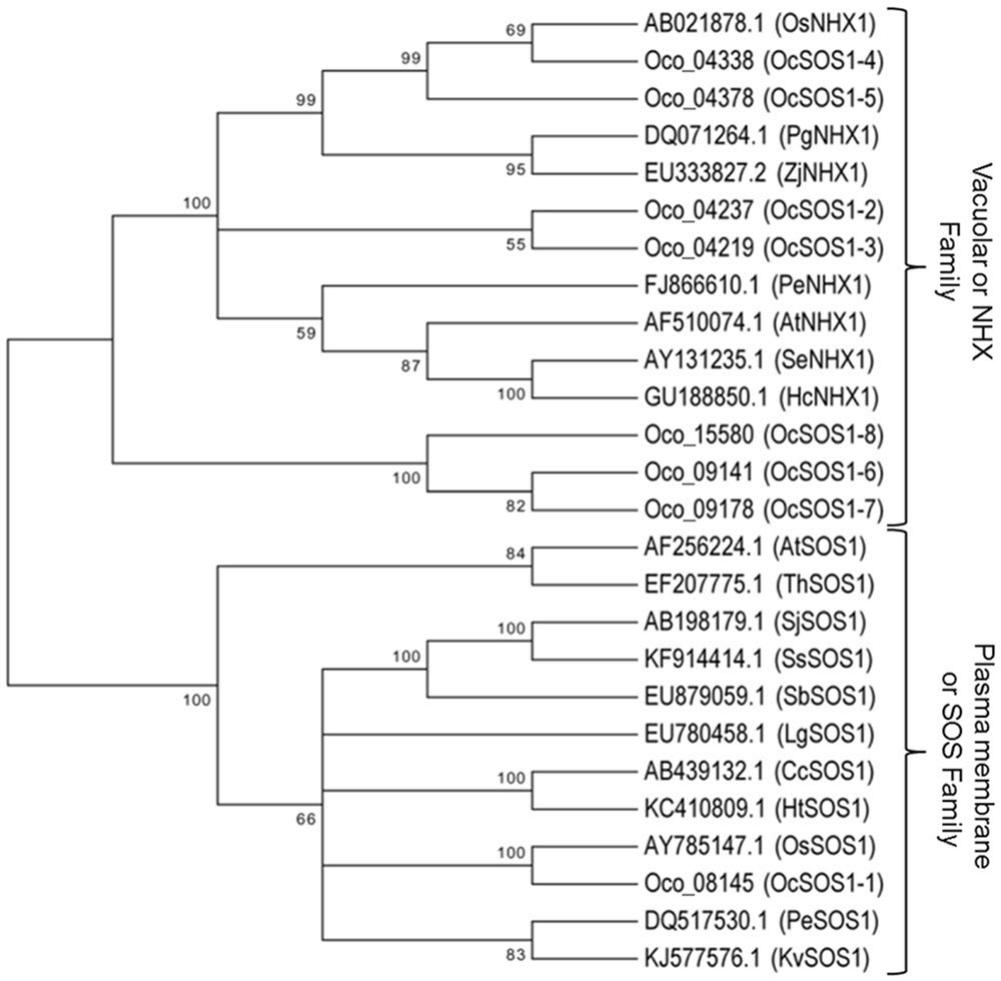
The phylogenetic analysis of *OcSOS1* genes with some other representative plant species to classify the identified 8 *SOS1* genes in to plasma membrane or vacuolar Na^+^/H^+^ antiporter genes.

There are 3 main genes in the SOS (salt overly sensitive) signal pathway, i.e. *SOS1*, *SOS2* and *SOS3* but as *SOS2* gets activated by *SOS3* after interaction, and their complex is required to fully activate *SOS1*, we focused on the identification of *SOS1* gene, a set of genes with most important role in plant salt-tolerance, to get an overlay of the abundance of *SOS* pathway related genes in the assembled genome^47–50^. Moreover *O*. *coarctata* is halophytic in nature and *SOS1* was found to have the activity needed for halophytic characteristic as reported in halophytic dicot *T*. *salsuginea*^51^. *SOS1* is one among the very few genes that required for plant salt tolerance which encodes for “plasma membrane Na^+^/H^+^ antiporter”, which not only plays an important role in germination but more importantly aid in the growth of plants in saline conditions^52^. So we focused on “Na^+^/H^+^ antiporter” domain or keyword based strategy to identify *SOS1* genes, as described in methodology. We identified a total of 8 *SOS1* genes in *O*. *coarctata* genome either by homology search against known *SOS1* genes or BLAST based function annotation of predicted genes and with the presence of “Na^+^/H^+^ antiporter or exchanger family” domain. To classify these eight *SOS1* genes in to plasma membrane Na^+^/H^+^ or vacuolar Na^+^/H^+^ antiporter gene, we have chosen 18 *SOS1* genes as used by Chen *et al*.^53^ and are available at NCBI with Accession numbers as: AF256224, AF510074, AB439132, GU188850, KC410809, KJ577576, EU780458, AY785147, AB021878, DQ517530, FJ866610, DQ071264, EU879059, AY131235, AB198179, KF914414, EF207775, EU333827, representing 11 plasma membrane and 7 vacuolar Na^+^/H^+^ antiporter genes from different plant species including *A*. *thaliana, Chrysanthemum crassum, Halostachys caspica, Helianthus tuberosus, Kosteletzkya virginica, Limonium gmelinii, O*. *sativa* ssp. *japonica, Populus euphratica, Pennisetum glaucum, Salicornia brachiata, Salicornia europaea, Suaeda japonica, S. salsa, T*. *halophile* and *Zoysia japonica*. A multiple sequence alignment and phylogenetic analysis was performed between these sequences and the close analysis of so obtained Neighbour Joining (NJ) tree suggested that only 1 of the 8 *SOS1* genes (*OcSOS1-1*) clustered with plasma-membrane Na^+^/H^+^ antiporters of other plant species while rest of the 7*SOS1* genes (*OcSOS1-2,3,4,5,6,7,8*) were part of the cluster having vacuolar Na^+^/H^+^ antiporters from other plant species (Fig. 3). But in both clusters, these genes were closely related with *SOS1* homologs of *O*. *sativa* ssp. *japonica*. Three of these identified *SOS1* genes i.e. *OcSOS1-6, OcSOS1-7* and *OcSOS1-8* could be the newly evolving ones as they showed a very low or no homology against known SOS1 genes and were present in a separate cluster in the obtained tree.

### Analysis of repetitive elements, SSRs and ncRNAs

A total of 113,352,647 bp (19.89%) of the *O*. *coarctata* genome was estimated to consist of repeated sequences (Table 1). We were able to identify 129,734 (14.95%) retrotransposons (class I), 79,468 (3.53%) DNA transposons (class II) and 3,092 (0.16%) unclassified elements in the genome (Supplemental Table S10).Among retroelements, 14.48% (82.53 Mb) of genome accounts for LTR retrotransposons and just 0.47% for Non-LTRs (SINEs and LINEs). The copia and gypsy are the two main components of LTR retrotransposons accounting 8.24% (46.94 Mb) and 6.04% (34.42 Mb) of the assembled genome, respectively. With LTR_FINDER, we have identified 218 full length LTR retrotransposons in 142 scaffolds (out of total 58362 scaffolds) comprising of a total of 1.30 Mb which accounts just 0.23% of *O*. *coarctata* genome (Supplemental Table S10).

A total of 230,968 SSRs were obtained across genome with an average frequency of one SSR per 2.46 kb. Out of total 58,362 scaffolds, 12,926 (22.15%) were found to contain SSR and 5,171 of these had more than one SSR (Supplemental Table S11). Among these identified SSRs, tetranucleotides (27.64%) were highest followed by trinucleotides (26.66%), mono- (19.99%), di- (16.79%), penta- (5.92%) and hexa-nucleotides (3.00%) (Fig S5a). For mono-, di- and tri- types of SSRs A/T, AT/TA, and CCG/CGG, were the motifs with highest percentages of 16.86%, 10.28% and 7.54%, respectively (Fig S5b).

Non-coding RNA genes have important regulatory roles in a number of plant phenomenons like chromosomal silencing, regulating the transcription process, developmental control, and various stress-responses^54^. Different types of ncRNAs were identified in *O*. *coarctata* genome included miRNA, rRNA, snRNA, tRNA and snoRNA (Supplemental Table S12). The *O*. *coarctata* genome was found to have 200 miRNA genes, 118 snRNA genes and 3,110 rRNA (LSU, SSU, 5_8S_rRNA and 5S_rRNA) genes encoded by “cmsearch” module of the INFERNAL package using the relevant covariance model from Rfam. The tRNAScan-SE algorithms, as applied with default parameters to the *O*. *coarctata* genome assembly, resulted in the identification of 900 putative tRNAs in the *O*. *coarctata* assembly. The snoRNA is a small RNA molecule that leads the chemical modifications of other RNAs, including rRNAs, tRNAs and snRNAs. In total 1,184 snoRNA were identified in *O*. *coarctata* genome.

### Single copy genes: Phylogenetic analysis of *O*. *coarctata* along with other 11 *Oryza* species

**Figure 4:**
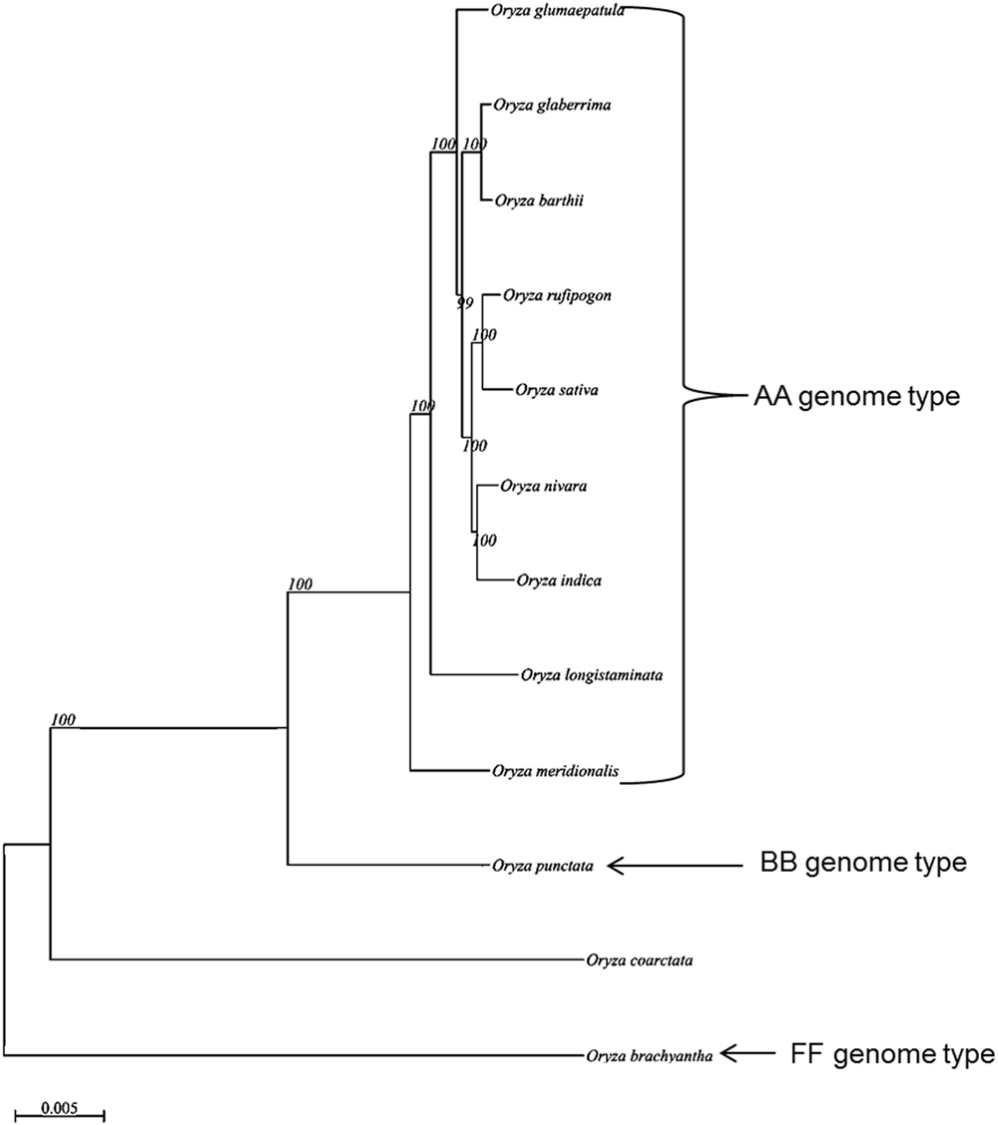
Tree topology of *O*. *coarctata* along with 11 other Oryza species based on the 170 clusters derived from 1:1 single-copy orthologous genes to depict the phylogeny at genome level to have an estimate of evolution of *O*. *coarctata* along with AA, BB and FF genome types.

In order to perform the genome level phylogenetic analysis of *O*. *coarctata* along with other 11 *Oryza* species, a total of 194,069 unique clusters were formed with a cut-off of 90% for coverage and similarity from their protein sequences. The cluster analysis resulted in total of 170 putative single copy gene clusters. These single copy proteins from the 12 genomes were considered for phylogenetic analysis. The tree generated with these single-copy orthologous genes placed *O*. *coarctata* as an individual clade and was found consistent with the species-tree obtained from the TimeTree database^55^, which generally used to retrieved the divergence times among species. A consensus analysis based on both the tree depict that FF genome (*O*. *brachyantha*) diverged about 15 million years ago (MYA) followed by *O*. *coarctata* around 10 MYA and the BB genome (*O*. *punctata*) about 6 MYA from the AA genome types. Among AA type genomes, it seems that Australian species, *O*. *meridionlais* was first to diverge from other AA genome species (South-American, Asian and African) and that happened somewhere around 2–3 MYA (Fig. 4).

### Synteny analysis

For representation of homologous blocks in synteny plots, unplaced scaffolds from each reference were concatenated and represented as single contig (“Un”) and the contigs with less than 1MB size in our assembled genome were concatenated and are represented as a single scaffold (“Unplaced”) (Supplemental Fig. S6). In the synteny analysis of *O*. *coarctata* with three of the dicot species including *A*.*thaliana*, *E*. *salsugineum* and *S*. *parvula*,high numbers of syntenic blocks were found with just 1 scaffold (S128; 1,335,219 bp) of *O*. *coarctata* having no collinear or syntenic block with any of these three species (Supplemental Fig. S6a–c). On the other hand, in spite of large genome size and more number of chromosomes in *O*. *sativa* ssp. *japonica*, there were 8 scaffolds of *O*. *coarctata* showing no collinearity or synteny with *O*. *sativa* ssp. *japonica* (Supplemental Fig. S6d) indicating that these scaffolds, which account for 12.52 Mb i.e. 2.19% of *O*. *coarctata* genome, might have some possible roles in halophytic adaptation of this species.

### Analysis of chloroplast genome

**Figure 5:**
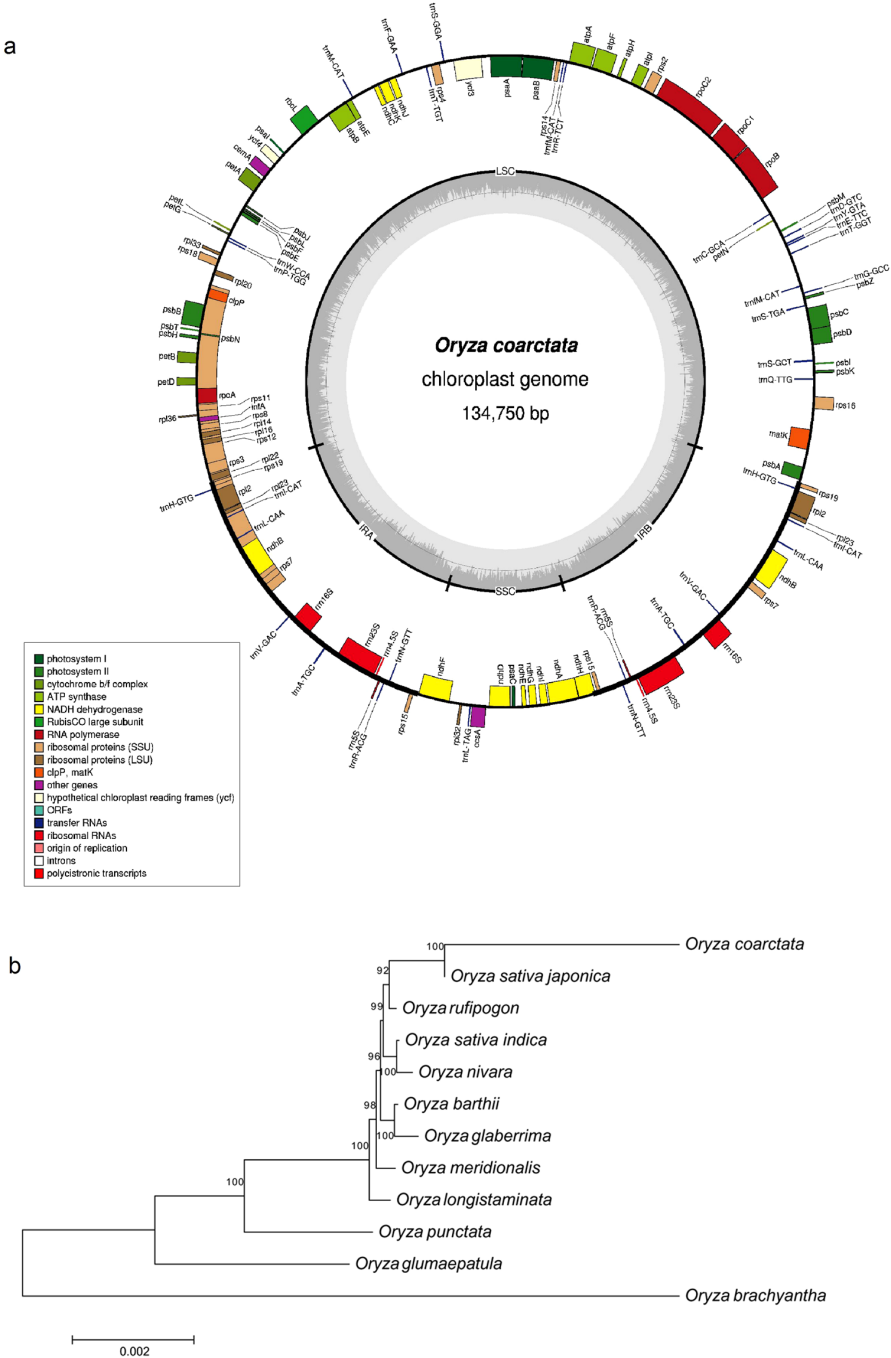
Genome organization and phylogeny of chloroplast genome of *O*. *coarctata*. (**a**) the chloroplast genome organization showing the genes transcribed clockwise (drawn inside the circle) and counter clockwise (drawn outside) with different gene functional groups are colour coded. (**b**) phylogenetic relationship of 12 *Oryza* species based upon whole choloroplast genome.

The chloroplast genome exists as circular molecule in angiosperms with size ranging from 120 to 160 kb in length^56^. We used 11 chloroplast sequences of *Oryza* species with size ranging from 134,558 bp to 135,525 bp as reference (Supplemental Table S1). Chloroplast reads were extracted from Nanopore data by BLAST search and assembled by guidance-based assembly into a circular contig of 134,750 bp length. It has a typical and standard quadripartite plant chloroplast structure with comparable regions of ~20.8 kb, ~80.8 kb and ~12.3 kb inverted repeats (IR), large single copy (LSC) and small single copy (SSC) regions, respectively with that of other *Oryza* species^56–58^. These four junctions were further confirmed and validated by PCR amplification (Supplemental Table S2; Fig. S7). While the IRa and IRb spanned for 20,800 bp region of assembled chloroplast, the LSC and SSC regions covered 80,816 bp and 12,334 bp, respectively (Fig. 5a and Supplemental Table S13). There were 82 protein coding genes, 33 tRNA genes and 8 rRNA genes, making a total of 123 genes with 18 genes as one copy in each of the 2 IR regions (Supplemental Table S13). In total, it has 43 genes involved in photosynthesis and divided in 6 different classes (Supplemental Table S14). No *ycf1* gene was found in this newly assembled chloroplast which supports the early loss of this gene in *Poaceae* family^58^. The phylogenetic analysis with chloroplast genomes of 11 other *Oryza* species infer that this newly assembled chloroplast genome is close to *japonica* subgroup of *Oryza* and is a part of the clade consisting of AA genome type while the BB and FF genome type, i.e. *punctata* and *brachyantha*, respectively were representing single different clades each (Fig. 5b).

### Analysis of mitochondrial genome

**Figure 6:**
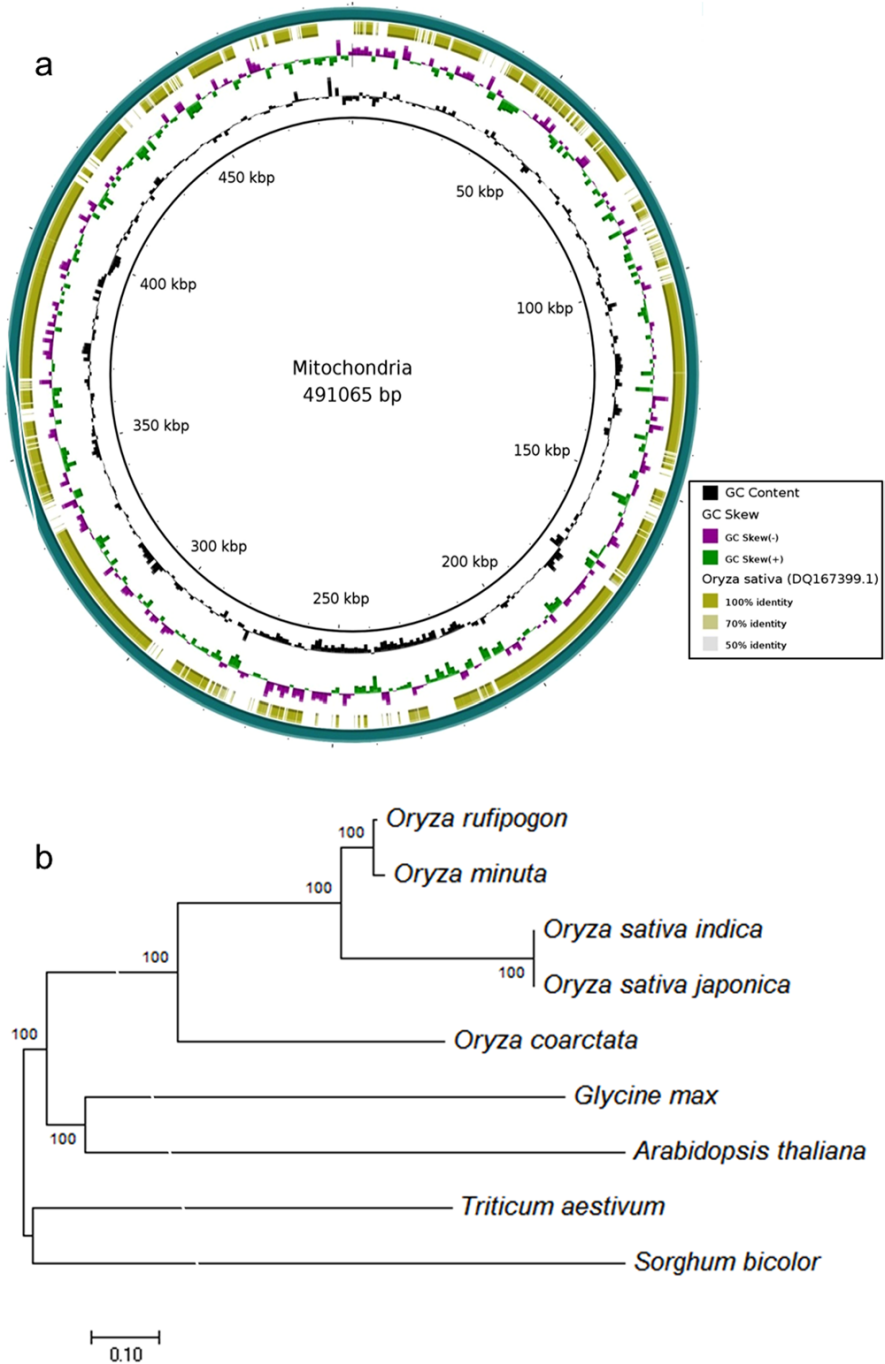
Assembly and phylogeny of mitochondrial genome of *O*. *coarctata*. (**a**) Circular representation of the *O*. *coarctata* mitochondrial genome showing GC content and identity with reference mitochondrial genome of *O*. *sativa* ssp. *japonica* at each base level. (**b**) Phylogenetic relationship of *O*. *coarctata* along with other species from Poaceae family and some other monocot and dicot species at mitochondrial genome level.

The assembled mitochondrial genome is 491,065 bp long (Fig. 6a) with 43.07% of GC content comprising of 46 protein coding genes, 37 tRNA genes and 4 rRNA genes (Supplemental Table S15). The annotation also confirm the presence of 4 genes related to functional category ‘Origins of replication’, making a total of 91 genes in the mitochondrial genome with a total of 48,960 bp (i.e.9.97% of assembled mitochondrial genome). The genome organization is comparable with available mitochondrial genomes of 4 other *Oryza* species with almost similar GC content and same range of protein coding genes, tRNA genes and rRNAgenes^59^. The annotated 46 protein coding genes here, included 25 genes related to the production of ATP synthase and the electron transport chain with 9, 1, 3 and 5 subunits of complex I (*nad1-9*), III (*cob*), IV (*cox1-3*) and V (*atp1,4,6,8,9*), respectively (Supplemental Table S16). Besides, there were 11 ribosomal proteins and only a single maturase (*mat-r*) gene.

To understand the evolutionary relationship of *O*. *coarctata*, at mitochondrial level, with other *Oryza* species, the mitochondrial genome sequences of 4 members of *Poaceae* family (*O*. *sativa* ssp. *japonica, O*. *sativa* ssp. *indica, O*. *rufipogon* and *O*. *minuta*), 2 other monocot species (*T*. *aestivum* and *S*. *bicolor*) and 2 dicot species (*G*. *max* and *A*. *thaliana*) were aligned to the newly assembled mitochondria genome of *O*. *coarctata* to construct a NJ tree (Fig. 6b). On one hand, this phylogenetic analysis with NJ tree clearly separates *Poaceae* family members from other 4 species included in the analysis and on the other hand, it placed *O*. *rufipogon* and *O*. *minuta* in one clade while *O*. *sativa* ssp. *japonica* and *O*. *sativa* ssp. *indica* in other clade as reported earlier by researchers^60^. But most importantly, we found *O*. *coarctata* in a group of *Poaceae* family, but in a different clade and closely related to the reference genome i.e. *O*. *sativa* ssp. *japonica*.

## Discussion

We report first time the draft genome sequence of a monocot halophyte, *O*. *coarctata* which provides an excellent platform for exploring the genetic potential of this wild species of rice. Our flow cytometry based results indicated the genome size be of 665 Mb, which is much closer to the previously reported estimated size of 771 Mb^61^. The results demonstrate several other interesting biological aspects and features of the genome. The Nanopore long reads significantly improved almost all the metrics related to quality of assembly, including reduced number of scaffolds, large size of the scaffolds (upto 7.85 Mb), a significant N50 (1.85 Mb) or L50 value, a good range of mean and median lengths (9.76 kb) along with a high percentage of genome completeness measure (upto 97.08%) based on the presence of core orthologous genes and hence contributed in the best representation of genome. It also helped us to retrieve the complete set of chloroplast genome sequence. To date, this is the only largest sequenced genome of any halophyte with the deepest sequencing coverage of 250.66-fold, considering the available sequenced halophytic genome such as *T*. *parvula*, *T*. *salsuginea* and/or *E*. *salsugineum* (Supplemental Table S17). A total of 33,627 predicted protein-coding genes that we discovered here, are the highest among sequenced halophytes and are comparable with other *Oryza* species. A 22.33% of these genes were found specific and unique to *O*. *coarctata* but rest were showing a good homology with rice (domesticated and AA type) and *O*. *brachyantha* (wild and FF type). The small genomes of chloroplast and mitochondria aid well in understanding the evolutionary background of *O*. *coarctata* in *Oryza* genus and among with AA, BB and FF genome type and supports the conservative nature of chloroplast and mitochondrial genomes within the taxon. Further, a phylogenetic analysis based on the single copy genes among *Oryza* species pointing towards the existence of *O*. *coarctata* genome somewhere between the divergence of FF and BB genome form AA genome. It could be true as assumed by researchers^5,13,62,63^ but needs to be validated with further study.

Further, when compared with rice, there were found 123 GO terms including “response to salt-stress”, “abscisic acid”, “biotic stress”, “desiccation”, “defence response to fungus”, etc., under GO category “response to stimulus”, exclusively present in *O*. *coarctata* but completely absent or present in low number in rice favouring the high salinity adaptation of this species. Moreover the presence of 8 *SOS1* gene copies in *O*. *coarctata* mayfavours and ads up to its salinity tolerance mechanism as *SOS1*contribute to salt tolerance by pumping the sodium ions out of the cells once activated^50^. Even the total identified (4,916) and stress-responsive TFs (1,440) were much larger in number than those found in rice (2,478 and 1,408, respectively) in earlier reports^64^. *Oryza coarctata*, even being a tetraploid, is found to have low repetitive content which is an exception and similar results were found reported by earlier researchers^65^. Although 82.53 Mb (14.48%) of genome consists of LTR retrotransposons but only 1.30 Mb (i.e. 0.23% of genome) of them were found as full-length LTRs, suggesting the probably high occurrences of recombination or deletion events post retrotransposition^66^. Comparatively a little higher synteny was found for *O*. *coarctata* with *E*. *salsugineum* and *S*. *parvula* as compared to rice, which could be due to the halophytic nature of these species indicating that, this genome sequence offers advantages as an ideal system for functional genomics to understand the salinity tolerance mechanisms of monocots as rest halophyte sequenced genome are dicot species. More importantly, our genome information will complement the I-OMAP project along with already existing genomic resources of different wild and cultivated *Oryza* species and will aid in both the genome level and the salinity based comparative genomics, evolutionary studies and extension of gene pool for improvement in cultivated rice.

## Electronic supplementary material

Supplementary files
