# Supplementary material for: Draft genome sequence of first monocot-halophytic species Oryza coarctata reveals stress-specific genes
Source: Sci Rep. 2018 Sep 12;8:13698. doi: 10.1038/s41598-018-31518-y (PMC6135824; doi:10.1038/s41598-018-31518-y)
Supplement: Supplementary file 1 — Supplementary files [file 41598_2018_31518_MOESM1_ESM.pdf]

Supplemental Information

Draft genome sequence of first monocot-halophytic species *Oryza coarctata* reveals stress-specific genes

Tapan Kumar Mondal*#, Hukam Chand Rawal#, Soni Chowrasia, Deepti Varshney, Alok Panda, Abhishek Majumdar, Harmeet Kaur, Kishor Gaikwad, Tilak Raj Sharma, Nagendra Kumar Singh.

ICAR-National Research Centre on Plant Biotechnology, Pusa, New Delhi-110012, India

*corresponding author (mondaltk@rediffmail.com) ; #contributed equally.

Table of contents

Supplementary figures: Figure S1 to S7	Page 1 to 7
Supplementary tables: Table S1 to S16	Page 8 to 19

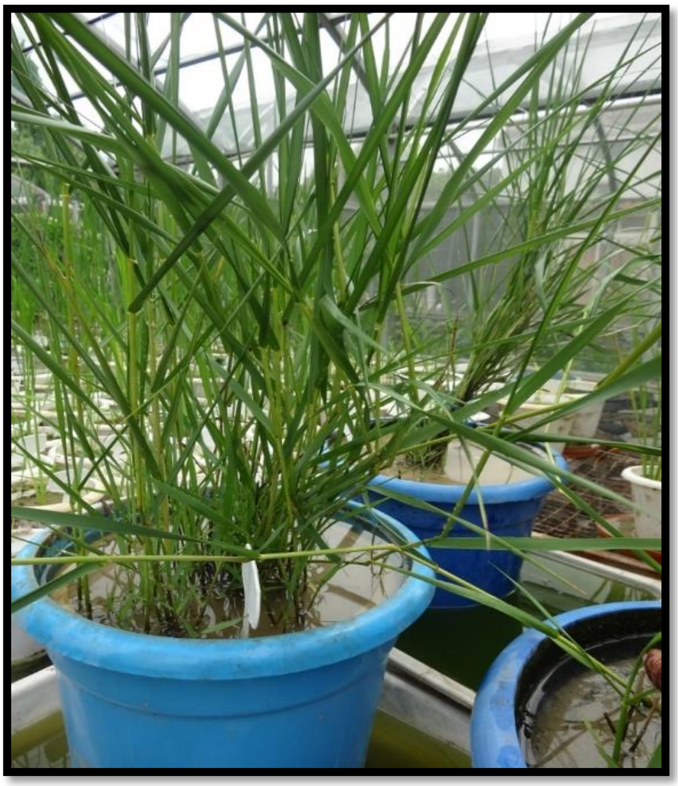

a

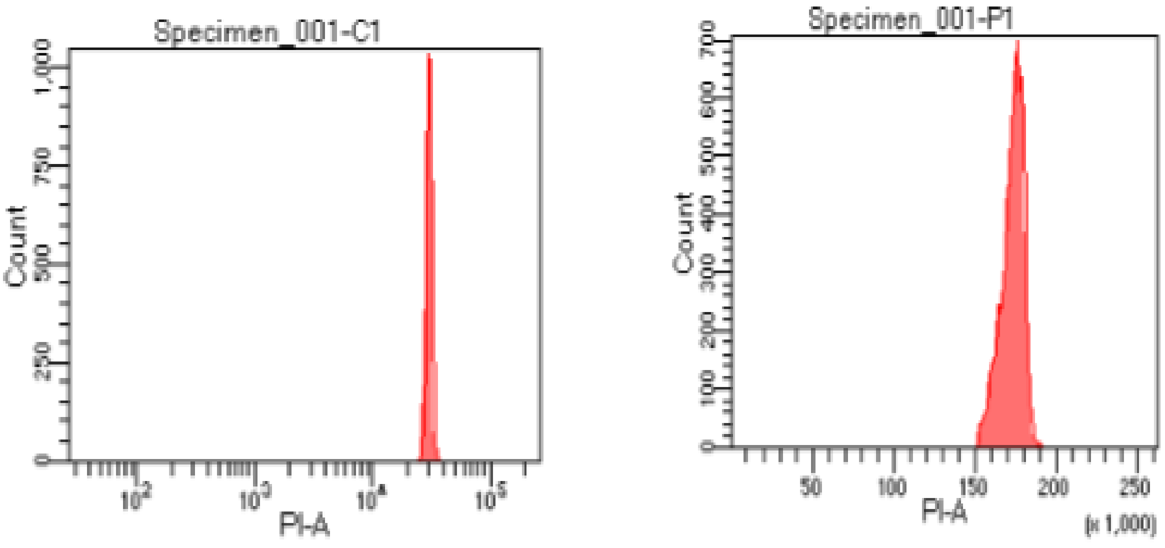

P. sativum ***O. coarctata***

b

Figure S1a. Pot grown young *O. coarctata* plants, leaf of which was used for genome sequencing, **S1b**. Flow cytometry results revealed the estimated genome size for *O. coarctata* as to be of 665 Mb with *Pisium sativum* was used as standard. X axis and Y axis represents relative DNA content and relative fluorescence intensity

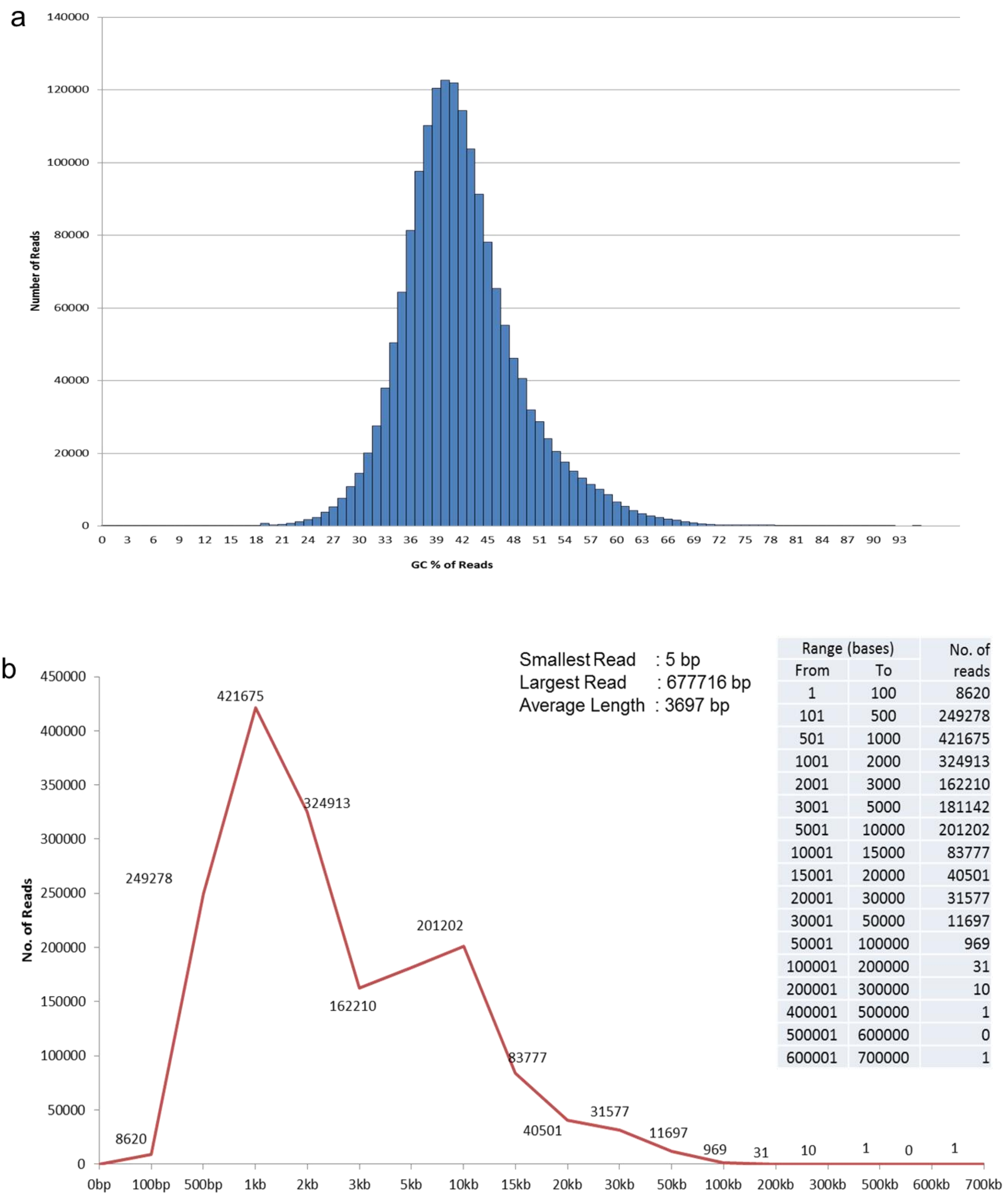

Range (bases) From	To	No. of reads
1	100	8620
101	500	249278
501	1000	421675
1001	2000	324913
2001	3000	162210
3001	5000	181142
5001	10000	201202
10001	15000	83777
15001	20000	40501
20001	30000	31577
30001	50000	11697
50001	100000	969
100001	200000	31
200001	300000	10
400001	500000	1
500001	600000	0
600001	700000	1

Figure S2a. GC content in Nanopore reads; **S2b.** Read length distribution of Nanopore reads

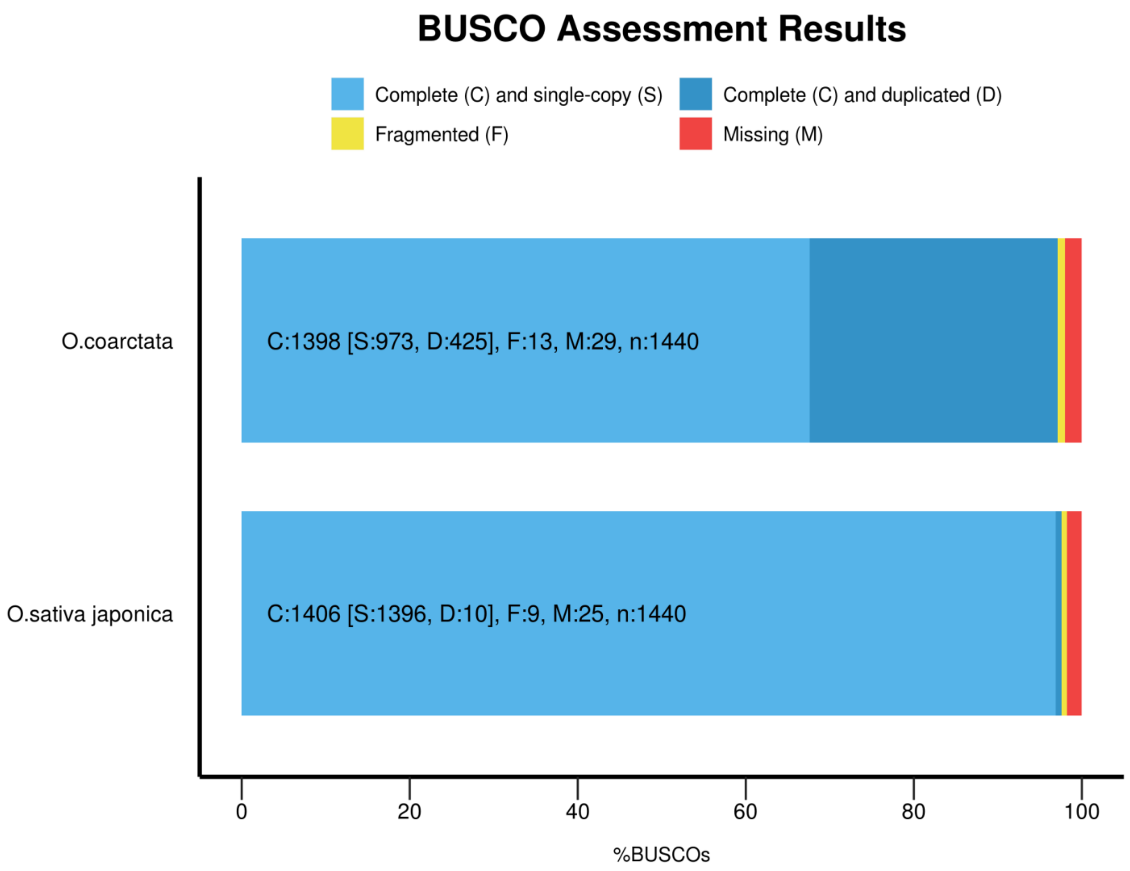

Figure S3. Completeness of *O. coarctata* and *O. sativa japonica* genome assemblies based on BUSCO analysis.

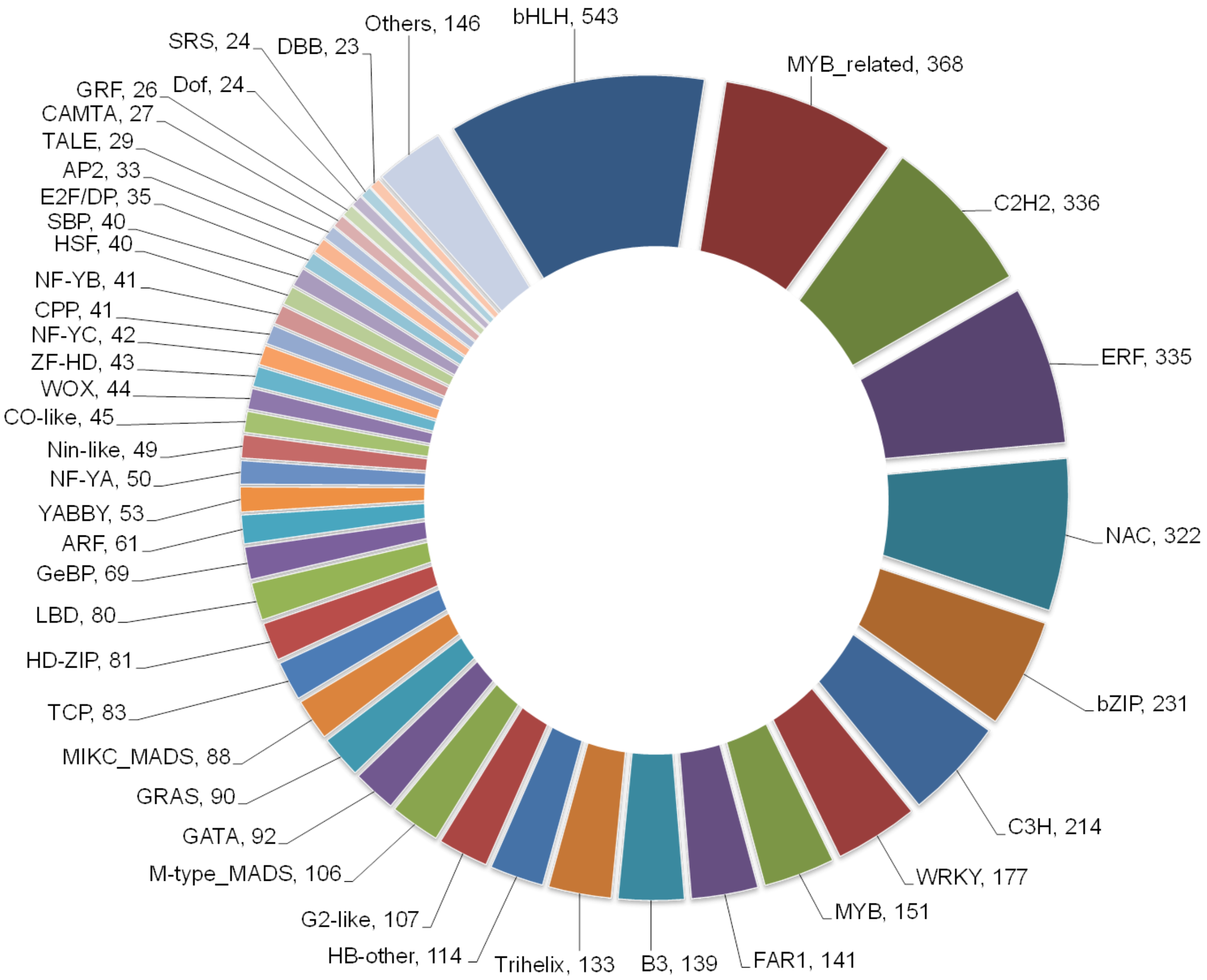

Figure S4. Transcription factor related genes in *O. coarctata* genome with high abundance of the stress-response related categories like MYB, MYB_related, NAC, bZIP, WRKY, HB, HSF and AP.

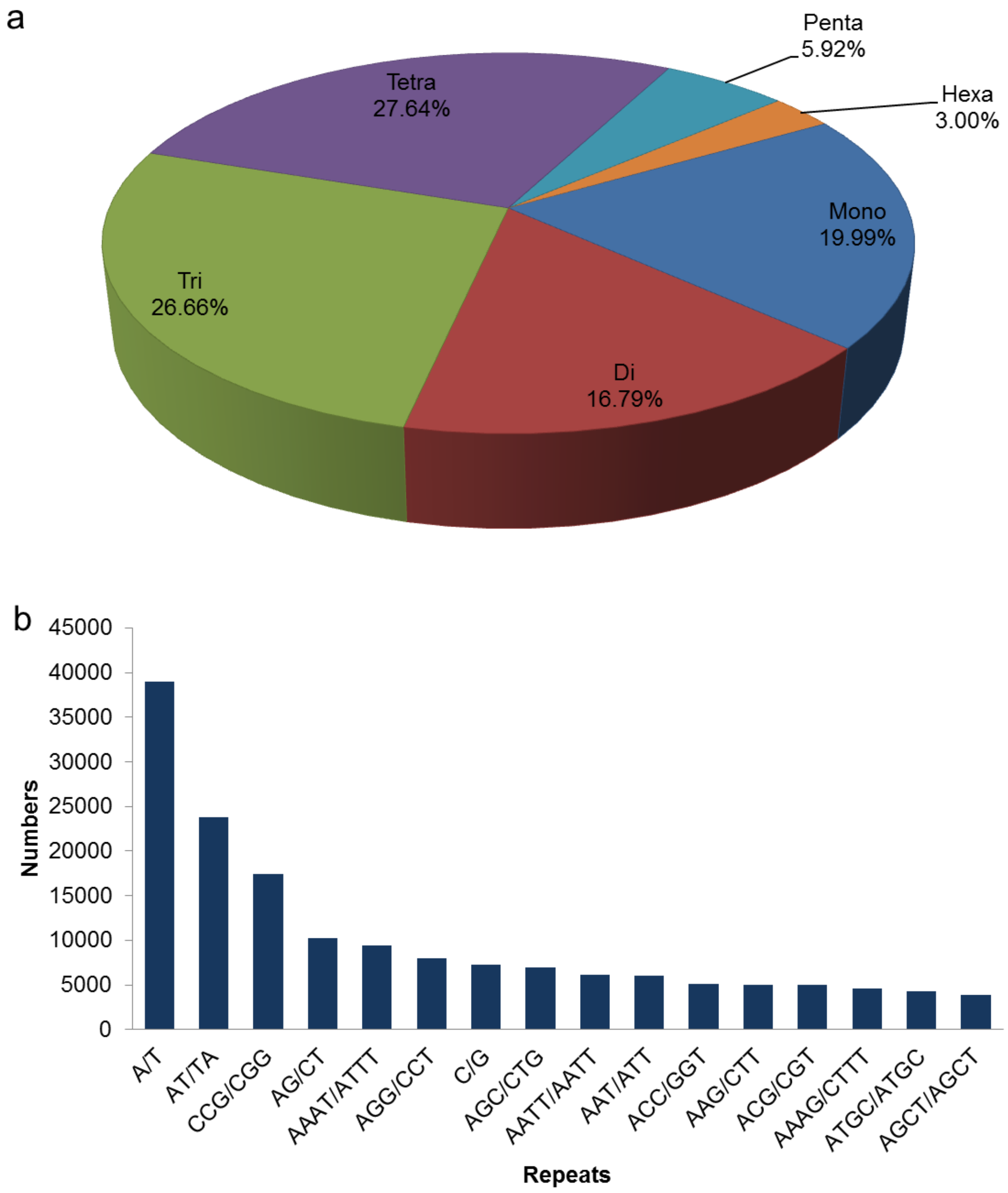

Figure S5a. Percentage distribution of identified SSRs into mono-, di-, tri-,tetra-, penta- and hexa-nucleotides; **S5b.** Distribution of mono-, di- and tri- types of SSRs

a

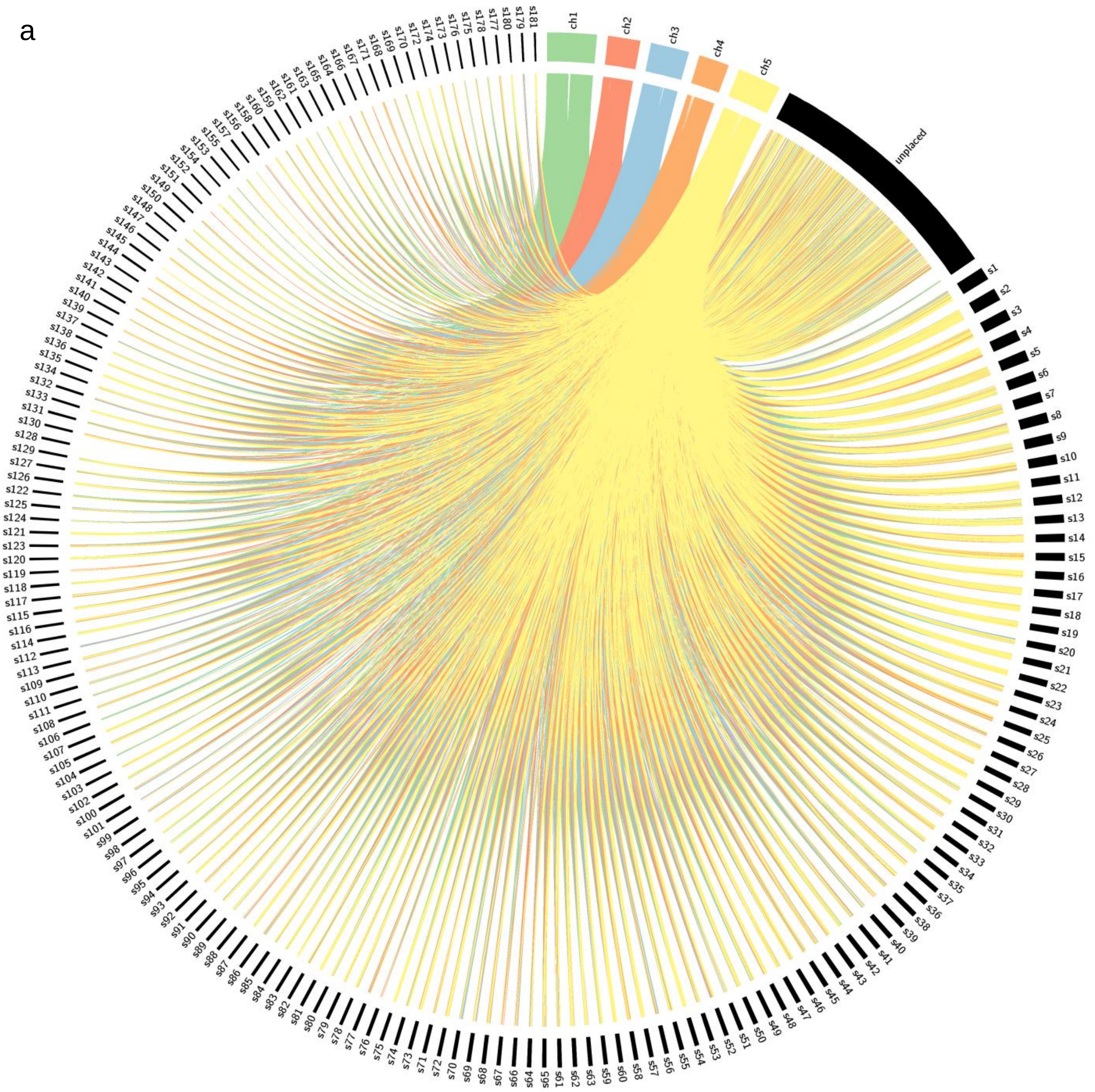

Figure S6a. Synteny plots showing homologous blocks between *O. coarctata* and *A. thaliana*

b

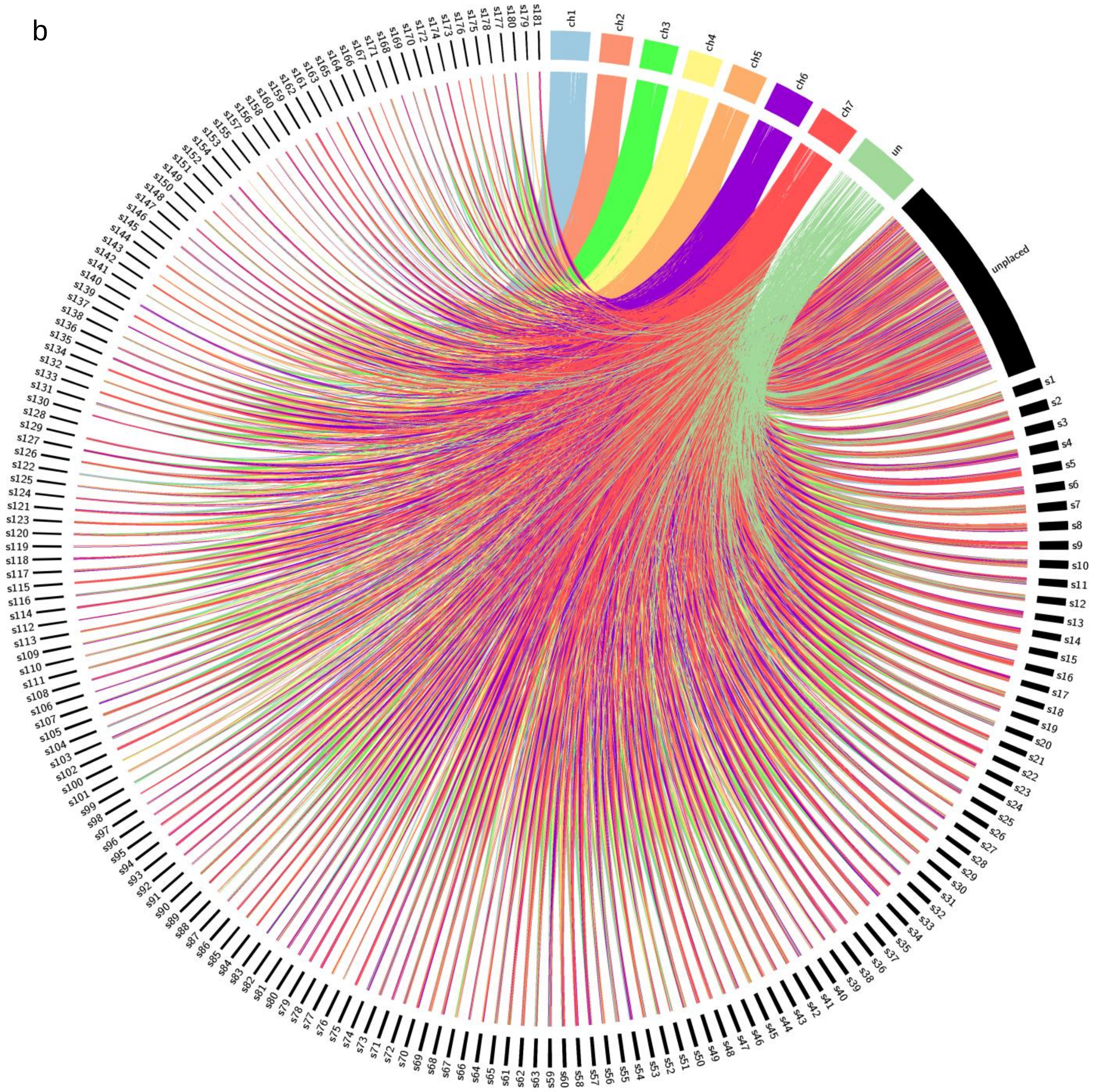

Figure S6b. Synteny plots showing homologous blocks between *O. coarctata* and *E. salsugineum*

c

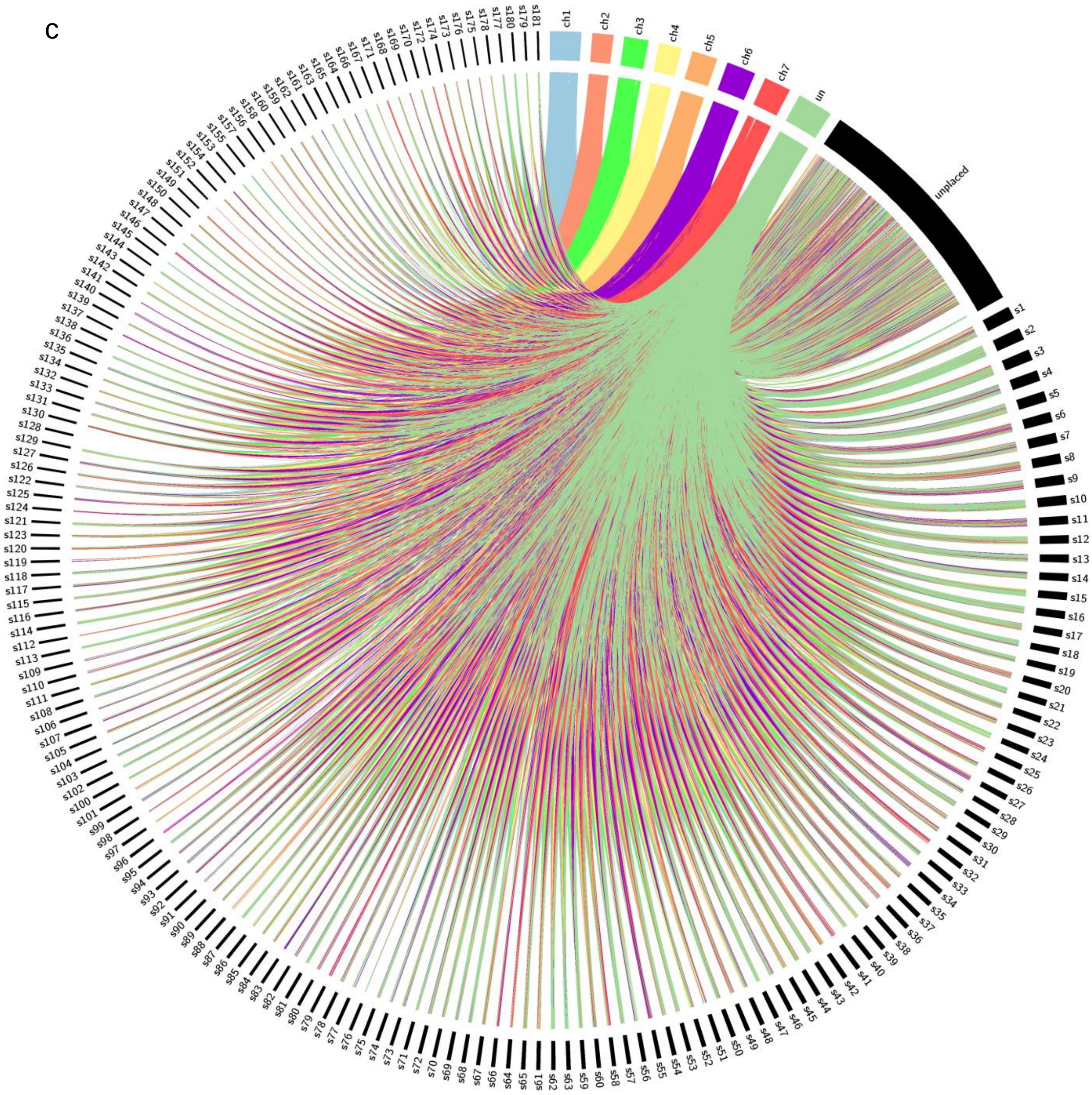

Figure S6c. Synteny plots showing homologous blocks between *O. coarctata* and *S. parvula*

d

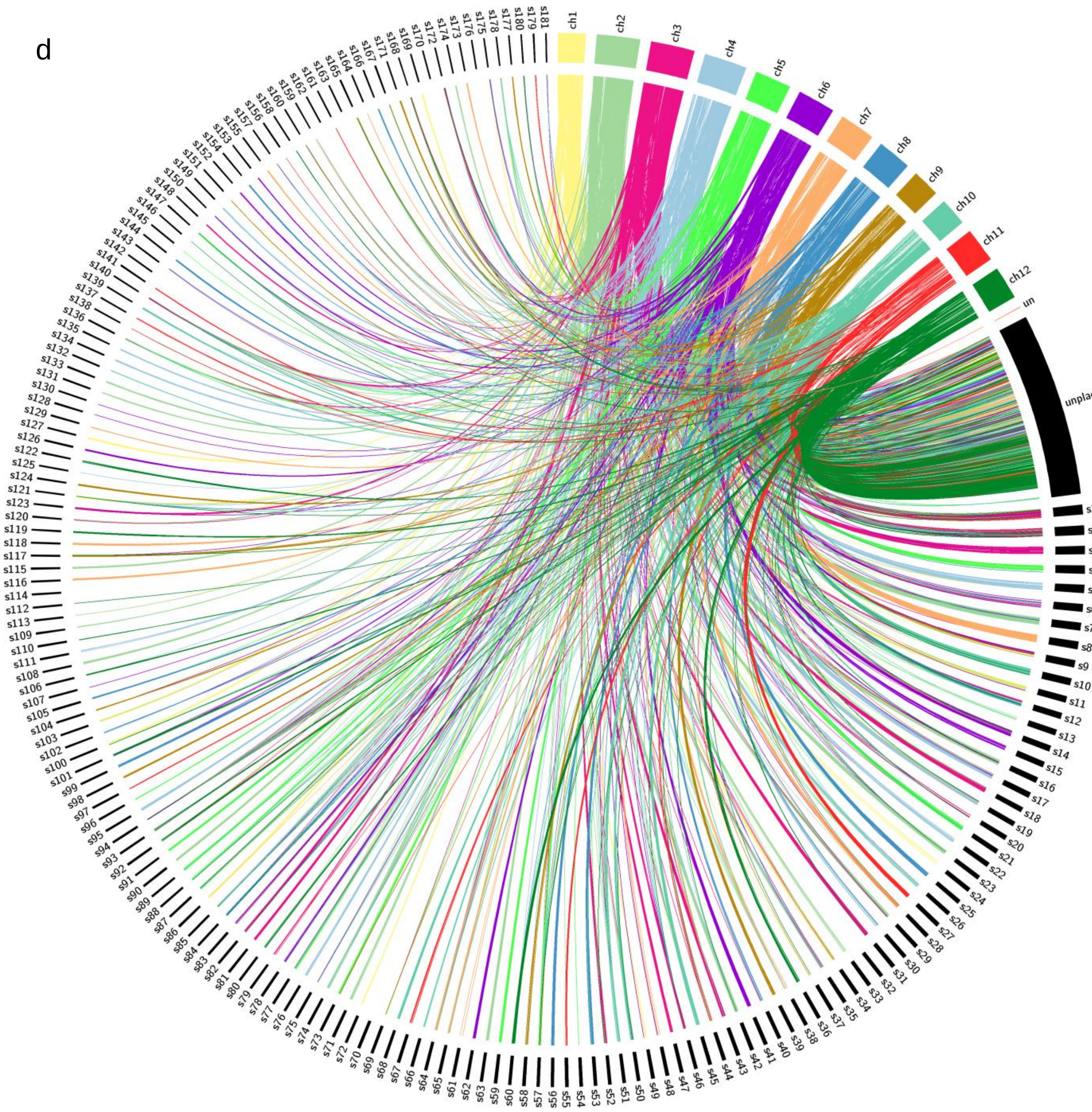

Figure S6. Synteny plots showing homologous blocks between *O. coarctata* and (**a**) *A. thaliana*, (**b**) *E. salsugineum*, (**c**) *S. parvula* and (**d**) *O. sativa japonica*. Each line link one homologous gene pair while the line colour representing each chromosome set of reference genome. The 181 linkage groups of *O. coarctata* are prefixed with "S" followed by the scaffold numbers, and the reference genomes chromosomes are designated with "ch" followed by their chromosome numbers.

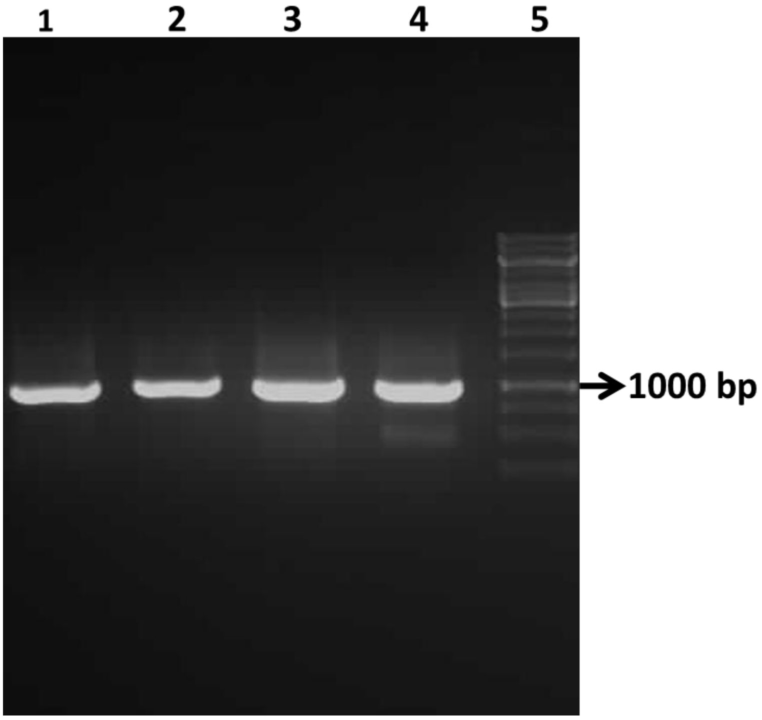

Figure S7. PCR validation of the chloroplast genome of *O. coarctata*. The agarose gel electrophoresis (1.5%) of the PCR amplified products of the four chloroplast genome junctions. The lane 1 represents the Junction of LSC/IRa; lane 2 represents Junction of IRa/SSC; lane 3 represents Junction of SSC/IRb ;lane 4 represents Junction of IRb/SSC and lane 5 represents 1 kbp (kilobase pair) ladder.

Table S1. Different Data used in this study:

Data Type	Database	Website Link/Accession no.
For comparative study between Oryza genus		
Protein sequences of 11 Oryza species	Gramene	http://www.gramene.org/
For Synteny analysis- Chromosome level assembly of 4 species		
Arabidopsis thaliana	Assembly Database at NCBI (https://www.ncbi.nlm.nih.gov/assembly/)	GCA_000001735.1
Schrenkiella parvula		GCA_000218505.1
Eutrema salsugineum		GCA_000325905.2
Oryza sativa japonica		GCA_001433935.1
Transcriptomic data used as expression evidence for gene prediction		
Transcriptome data (5 Samples) of *Oryza (Porteresia) coarctata*	SRA database at NCBI https://www.ncbi.nlm.nih.gov/sra	SRP019245
Chloroplast genomes of 11 *Oryza* species used as references for chloroplast assembly of *O. coarctata*		
Oryza sativa japonica	Nucleotide database at NCBI https://www.ncbi.nlm.nih.gov/nuccore/	X15901.1
Oryza rufipogon		KF359902.1
Oryza sativa indica		NC_008155.1
Oryza nivara		NC_005973.1
Oryza meridionalis		NC_016927.1
Oryza longistaminata		KM088024.1
Oryza punctata		KF359908.1
Oryza glumaepatula		KF359905.1
Oryza brachyantha		KF359917.1
Oryza barthii		KM088023.1
Oryza glaberrima		KF359903.1
Mitochondrial genomes used as references and in phylogeny for mitochondrial assembly of *O. coarctata*		
Oryza sativa japonica	Nucleotide database at NCBI https://www.ncbi.nlm.nih.gov/nuccore/	NC_011033.1
Oryza sativa indica		NC_007886.1
Oryza rufipogon		AP011076.1
Orya minuta		KU176938.1
Triticum aestivum		NC_007579.1
Sorghum bicolor		NC_008360.1
Glycine max		NC_020455.1
Arabidopsis thaliana		NC_001284.2

Table S2. List of pairs of primers used for PCR validation

O. coarctata plantlets were grown in glass house under 16 h light (25 °C)/ 8 h dark (16 °C) conditions. Fresh leaves from the plantlet were collected and total genomic DNA was isolated from 100 mg of leaves by using CTAB method. The residual RNA was removed by RNase I treatment (Thermo Fisher Scientific, USA) and integrity of DNA was checked in 0.8% agarose. *In silico* gene map of chloroplast genome of *O. coarctata* was designed. In order to validate the gene map, the primers were prepared from the four junctions between LSC/IRs and SSC/IRs of the chloroplast gene map. All primers were designed by using Vector NTI software (Lu et al., 2004). The primer sequences along with its Tm (^{0}C) and product length are provided in Table 1.

The four junctions of chloroplast genome were amplified using the polymerase of GoTaq® DNA Polymerase (Promega). The reaction mixture of 25 μ L was prepared for PCR amplification. The amplification of the LSC/IRs and SSC/IRs regions was performed in Agilent PCR: 5 min at 97 °C for denaturation; 35 cycles of 1 min at 95 °C (denaturation), 1 min at 59- 62°C (annealing), and 2 min at 72 °C (extension); and a final step of 10 min at 72 °C for extension. The PCR products were checked on 1% agarose and also send for sequences for confirmation.

Junctions	Sequence	Tm (^{0}C)	Product size(base pair)
Junction of LSC/IRa	F:GGTGATTTTGGGTTGTACCAAGTCTGAAAC<br>R: CGAGATATTGGACATCGAATGCCACTC	62	966
Junction of IRa/SSC	F:CAAGTATTCAGTTTCACCAATAAGATACGGAGAC<br>R: TTCCAAGAAGTTCTAATTCGTCCATAAATTC	59	990
Junction of SSC/IRb	F: CCCAATTTACAGCTTCTTCTCCGCTAA<br>R: GTTTCACCAATAAGATACGGAGACTTGCTTC	61	972
Junction of IRb/SSC	F: GGATTCACAATCCACTGCCTTGATCC<br>R: AGGAAACCACTGAAATGAATCTGCTAATGAG	62	948

Reference

Lu, G., Moriyama, E.N. (2004). Vector NTI, a balanced all-in-one sequence analysis suite. .*Brief. Bioinform*5, 378-388. doi: 10.1093/bib/5.4.378

Table S3. Genome coverage and sequencing reads statistics

S. No.	Reads Type	Genome coverage (x) using Estimated Genome size (~665 Mb)	High-Quality Processed Data	
			Reads	Bases
1.	PE	186.14	412,607,853	123,782,355,900
2.	2 Kb	16.73	37,093,166	11,127,949,800
3.	4 Kb	4.61	10,218,333	3,065,499,900
4.	6 Kb	16.89	37,439,915	11,231,974,500
5.	8 Kb	16.74	37,105,001	11,131,500,300
6.	Nanopore	9.55	1,717,607	6,350,953,369
	Total	250.66	536,181,875	166,690,233,769
PE – Illumina paired-end reads; 2 Kb, 4 Kb, 6 Kb and 8 Kb – Mate-pair reads with insert size of 2 Kb, 4 Kb, 6 Kb and 8 Kb, respectively.				

Table S4. Completeness of the genome assembly by CEGMA based analysis

Pathotypes	*O. coarctata*		Reference	
	Complete	Partial	Complete	Partial
No. of proteins	229	241	232	245
% Completeness	92.34	97.18	93.55	98.79
Total	611	727	426	492
Average	2.67	3.02	1.84	2.01
% Ortho	83.41	91.29	51.72	57.96
Normalized % Completeness	98.70	98.36	100	100

*Reference= *Oryza sativa* spp. *japonica* Nipponbare -IRGSP-1.0

Keys:

Prots = Number of 248 ultra-conserved CEGs present in genome

%Completeness = Percentage of 248 ultra-conserved CEGs present

Total = Total number of CEGs present including putative orthologs

Average = Average number of orthologs per CEG

%Ortho = Percentage of detected CEGS that have more than 1 ortholog

Normalized % = Completeness with respect to reference

Table S5. Number of genes present in 11 Oryza species genomes used for comparative study

S.No.	Species	Total	Range (aa)	Begins with M	Begins with M and >50 aa
1	*O. coarctata*	42284	4569-23	34851	33627
2	*O. sativa japonica*	42132	5342-14	32384	32218
3	*O. sativa indica*	40745	5024-32	40745	40701
4.	*O. rufipogon*	47441	6304-1	47235	47042
5.	*O. punctata*	41060	5284-1	40946	40803
6.	*O. nivara*	48360	5485-1	48163	47982
7.	*O. meridionalis*	43455	5440-1	43251	43059
8.	*O. longistaminata*	31686	5154-2	28237	27660
9.	*O. glumaepatula*	46893	5488-1	46723	46535
10.	*O. glaberrima*	33164	4738-23	28958	28869
11.	*O. brachyantha*	32037	5111-28	30420	30312
12.	*O. barthii*	41595	5436-1	41431	41260

Table S6. Statistics of genes per species in the plant gene database used for annotation (top 50 are shown)

S.No.	Species	Proteins
1	*Brassica napus*	113056
2	*Camelina sativa*	107566
3	*Gossypium hirsutum*	91320
4	*Nicotiana tabacum*	84981
5	*Helianthus annuus*	73959
6	*Glycine max*	71209
7	*Arabidopsis thaliana*	63768
8	*Chenopodium quinoa*	63172
9	*Raphanus sativus*	61234
10	*Malus domestica*	60654
11	*Zea mays*	59499
12	*Gossypium raimondii*	59094
13	*Hevea brasiliensis*	58083
14	*Medicago truncatula*	57753
15	*Arachis ipaensis*	57604
16	*Brassica oleracea var. oleracea*	56622
17	*Juglans regia*	55711
18	*Aegilops tauschii subsp. tauschii*	55630
19	*Lupinus angustifolius*	52833
20	*Arachis duranensis*	52811
21	*Brassica rapa*	52555
22	*Eucalyptus grandis*	52553
23	*Ipomoea nil*	51068
24	*Populus euphratica*	49783
25	*Nicotiana tomentosiformis*	49041
26	*Nicotiana sylvestris*	48312
27	*Musa acuminata subsp. malaccensis*	47720
28	*Gossypium arboreum*	47619
29	*Pyrus x bretschneideri*	47064
30	*Populus trichocarpa*	46066
31	*Capsicum annuum*	45495
32	*Oryza sativa Japonica Group*	44930
33	*Daucus carota subsp. sativus*	44587
34	*Nicotiana attenuata*	44494
35	*Manihot esculenta*	43333
36	*Elaeis guineensis*	41875
37	*Vitis vinifera*	41380
38	*Tarenaya hassleriana*	41179
39	*Sorghum bicolor*	39414
40	*Arabidopsis lyrata subsp. lyrata*	39157
41	*Cajanus cajan*	38966
42	*Phoenix dactylifera*	38567
43	*Solanum tuberosum*	38362

44	*Nelumbo nucifera*	38232
45	*Vigna angularis*	37747
46	*Ziziphus jujuba*	37646
47	*Asparagus officinalis*	36857
48	*Solanum lycopersicum*	36515
49	*Physcomitrella patens*	36066
50	*Ananas comosus*	35784

Table S7. Rice (IRGSP1.0) vs *O. coarctata* annotation analysis showing differences (of atleast 5) for number of genes with stress related GO terms.

GO-IDs	Gene counts		GO category (Function)
	O. coarctata	Rice	
GO:0009733	91	1	response to auxin
GO:0006950	94	42	response to stress
GO:0042744	11	0	hydrogen peroxide catabolic process
GO:0050832	10	0	defense response to fungus
GO:0009269	9	0	response to desiccation
GO:0006654	7	0	phosphatidic acid biosynthetic process
GO:0009607	7	0	response to biotic stimulus
GO:0048017	7	0	inositol lipid-mediated signaling
GO:0000724	6	0	double-strand break repair via homologous recombination
GO:0009737	6	0	response to abscisic acid
GO:0010112	6	0	regulation of systemic acquired resistance
GO:2000028	6	0	regulation of photoperiodism, flowering
GO:0009651	5	0	response to salt stress
GO:0009742	5	0	brassinosteroid mediated signaling pathway
GO:0009958	5	0	positive gravitropism

Table S8. Statistics of BLAST and annotation with Blast2GO

Data type	Genes
Predicted genes	33627
Annotated with Blast against Plant Genes DB	26569
Genes with assigned GO Terms	16357
Genes with InterProScan Match	30886
Genes with Enzyme Commission Classification	2,844
Genes with No Blast hit	7058
Genes with No GO terms	17270
Genes with No BLAST hits but GO terms	120
Genes with No BLAST hits but IPS match	4842
Genes with neither BLAST hits nor GO terms nor IPS match	2216

Table S9. Number of TF containing genes of *O. coarctata* shared across other species of plants

Organism Name	No. of genes shared
Oryza punctata	560
Oryza brachyantha	261
Malus domestica	209
Oryza meridionalis	186
Oryza sativa subsp. japonica	181
Fragaria vesca	163
Oryza longistaminata	150
Actinidia chinensis	148
Oryza glumaepatula	147
Oryza sativa subsp. indica	141
Leersia perrieri	136
Oryza nivara	111
Sisymbrium irio	110
Zoysia matrella	88
Oryza glaberrima	88
Fragaria ananassa	87
Dorcoceras hygrometricum	84
Phyllostachys heterocycla	83
Oryza rufipogon	82
Ipomoea trifida	81
Brassica napus	80
Oryza barthii	74
Musa acuminata	70
Daucus carota	70
Zoysia japonica	64
Gossypium hirsutum	63
Dichanthelium oligosanthes	60
Linum usitatissimum	59
Oropetium thomaeum	55
Raphanus sativus	51
Solanum melongena	50
Others	1124
Total	4916

Table S10. Repeat masking statistics of *O. coarctata* genome

TE Class	Family	Sub-family	No. of Elements	Length Occupied (bp)	% in the genome
Class I (Retroelements)	Non-LTR elements	SINEs:	1450	217932	0.04
		LINEs:	3826	2456111	0.43
	LTR elements	Ty1/Copia	47330	46943177	8.24
		Gypsy/DIRS1	73040	34417003	6.04
		Total LTR elements	124458	82529172	14.48
Total Retroelements[a]			129734	85203215	14.95
Class II (DNA transposons)	DNA transposons	hobo-Activator	4857	1305561	0.23
	DNA transposons	Tc1-IS630-Pogo	15111	2448176	0.43
	DNA transposons	Tourist/Harbinger	16361	2564908	0.45
Total DNA Transposons[b]			79468	20121450	3.53
Unclassified[c]			3092	892369	0.16
Total interspersed repeats [(a+b+c)]				106217034	18.63
Small RNA			1605	284272	0.05
Satellites			141	24922	0
Simple repeats			136349	5835615	1.02
Low complexity			23175	1153463	0.2
Total bases masked				113352647	19.89
Full length LTR retrotransposons			218	1308198	0.23

Table S11. Statistics of SSRs

Stats	Count
Total number of sequences examined	58362
Total size of examined sequences (bp)	569994164
Total number of identified SSRs	230968
Number of SSR containing sequences	12926
Number of sequences containing more than 1 SSR	5171
Number of compound SSRs	26494
Mono	46172
Di	38786
Tri	61568
Tetra	63849
Penta	13668
Hexa	6925

Table S12. Annotation of conserved non-coding RNA genes in *O. coarctata* genome

ncRNA Type	#Loci	Average length (bp)	Total length (bp)	Percentage of genome (%)
miRNA	200	135	27,018	0.00
tRNA	900	74	66,822	0.01
rRNA (LSU)	226	263	59,482	0.01
rRNA(SSU)	142	255	36,158	0.01
rRNA(5_8S_rRNA)	8	130	1,037	0.00
rRNA (5S_rRNA)	2,734	108	294,806	0.05
SnoRNA	1,184	119	140,685	0.02
snRNA	118	141	16618	0.00

Table S13. Features of the chloroplast genome of *O. coarctata*

Feature	Length (bp)	Number count
Genome	134750	1
LSC	80816	1
SSC	12334	1
Inverted Repeat (IR)Regions	20800	2 (IRa, IRb)
Protein Coding genes	59086	82
tRNA	2419	33
rRNA	9182	8

Table S14. List of genes present in the chloroplast genome of *O. coarctata*

**Category**	**Gene Name**
Photosystem I	*psaA,B,C,I*
Photosystem II	*psbA,B,C,D,E,F,H,I,J,K,L,M,N,T,Z*
Cytochrome b6/f	*petA,B,D,G,L,N*
ATP Synthase	*atpA,B,E,F,H,I*
Rubisco	*rbcL*
NADH-dehydrogenase	*ndhA,B*#*,C,D,E,F,G,H,I,J,K*
Large subunit ribosomal proteins	*rpl2*#*,14,16,20,22,23*#*,32,33,36*
Small subunit ribosomal proteins	*rps11, rps12*#*, rps14, rps15*#*, rps16, rps18, rps19*#*, rps2, rps3, rps4, rps7*#*, rps8*
DNA dependent RNA polymerase	*rpoA,B,C1,C2*
Other Proteins	*ccsA, cemA, clpP, infA, matK*
Proteins of unknown Function	*ycf3, ycf4*
Ribosomal RNAs	*rrn16S*#*, rrn23S*#*, rrn4.5S*#*, rrn5S*#
Transfer RNAs	*trnA-TGC*#*, trnC-GCA, trnD-GTC, trnE-TTC, trnF-GAA, trnfM-CAT, trnG-GCC, trnH-GTG*#*, trnI-CAT*#*, trnL-CAA*#*, trnL-TAG, trnM-CAT, trnN-GTT*#*, trnP-TGG, trnQ-TTG, trnR-ACG*#*, trnR-TCT, trnS-GCT, trnS-GGA, trnS-TGA, trnT-GGT, trnT-TGT, trnV-GAC*#*, trnW-CCA, trnY-GTA*

[#]Two gene copies in the IRs

Table S15. Features of the mitochondrial genome of *O. coarctata*

Features	Statistics
Genome size (bp)	491065
GC contents (%)	43.07
Total Genes	91
Protein Coding genes	46 (40206 bp)
tRNA	37 (2807 bp)
rRNA	4 (5671 bp)
Total Gene Length (bp)	48960 (9.97% of genome)

Table S16. Genetic contents in the mitochondria genome of *O. coarctata*

**Functional Group**	**Genes**
Complex I (NADH dehydrogenase subunits)	*nad1, nad2, nad3, nad4, nad4l, nad5, nad6, nad7, nad9*
Complex III (Apocytochrome b)	*cob*
Complex IV (Cytochrome c oxidase subunits)	*cox1, cox2, cox3*
Complex V (ATP synthase subunits)	*atp1, atp4, atp6_0, atp6_1, atp6_2, atp8, atp9_0, atp9_1*
Cytochrome C	*ccmB, ccmC, ccmFc, ccmFn*
Ribosomal large subunit	*rpl2, rpl5, rpl16*
Ribosomal small subunit	*rps1, rps2, rps3, rps4, rps7, rps12, rps13, rps19,*
Ribosomal RNAs (rRNA)	*rrnL, rrnS_0-a, rrnS_0-b, rrnS_1*
Transfer RNAs (tRNA)	*trnC_0(gca), trnC_1(gca), trnC_2(gca), trnD(gtc), trnF_0(gaa), trnF_1(gaa), trnH_0(gtg), trnH_1(gtg), trnH_2(gtg), trnK(ttt), trnL1(---), trnL2(caa), trnM_0(cat), trnM_1(cat), trnM_2(cat), trnM_3(cat), trnM_4(cat), trnM_5(cat), trnM_6(cat), trnM_7(cat), trnM_8(cat), trnN(gtt), trnP_0(tgg), trnP_1(tgg), trnP_2(tgg), trnQ(ttg), trnR(tct), trnS1_0(gct), trnS1_1(gct), trnS2_0(tga), trnS2_1(tga), trnS2_2(gga), trnV(gac), trnW_0(cca), trnW_1(cca), trnY_0(gta), trnY_1(gta)*
Origins of replication	*OH-a, OH-b, OH-c, OH-d*
Intron maturase	*mat-r*

Table S17. Summary of the sequenced halophyte genomes

Organism/species		*T. parvula* (*E. parvulum* or *S. parvula*)	*T. salsuginea* (*T. halophila*)	*E. salsugineum* (*T. halophila*)	*O. coarctata*
Dicot or monocot		Dicot	Dicot	Dicot	Monocot
Genome size	Expected	140	260	260	665
	Assembled	137.09	233.7	243.1	569.99
Coverage (X)		~50	~134	~8	250.66
Repetitive elements (%)		7.46%	52%	51.40%	19.89%
Protein-coding genes		30419	28457	26,531	33,627
Reference		Dassanayake et al., (2011)	Wu et al., (2012	Yang et al., (2013)	In the present study
